# Glycosphingolipid GM3 prevents albuminuria and podocytopathy induced by anti-nephrin antibody

**DOI:** 10.1038/s41598-022-20265-w

**Published:** 2022-09-26

**Authors:** Nagako Kawashima, Shokichi Naito, Hisatoshi Hanamatsu, Masaki Nagane, Yasuo Takeuchi, Jun-ichi Furukawa, Norimasa Iwasaki, Tadashi Yamashita, Ken-ichi Nakayama

**Affiliations:** 1 Department of Nephrology, School of Medicine, Kitasato University, 1-15-1 Kitasato, Minami, Sagamihara, Kanagawa 252-0374 Japan; 2 Department of Advanced Clinical Glycobiology, Faculty of Medicine and Graduate School of Medicine, Hokkaido University, Kita15jyo - Nishi7chome, Kita, Sapporo, Hokkaido 060-8638 Japan; 3 Department of Gastroenterology and Hepatology, Graduate School of Medicine, Hokkaido University, Kita15jyo-Nishi7chome, Kita, Sapporo, Hokkaido 060-8638 Japan; 4 Biochemistry, School of Veterinary Medicine, Azabu University, 1-17-71 Fuchinobe, Chuo, Sagamihara, Kanagawa 252-5201 Japan; 5 Institute for Glyco-Core Research (iGCORE), Nagoya University, 65 Tsurumai, Showa, Nagoya, Aichi 466-8550 Japan; 6 National Institute of Advanced Industrial Science and Technology (AIST), 1-1-1 Umezono, Tsukuba, Ibaraki 305-8560 Japan

**Keywords:** Glomerulus, Glycolipids, Glycobiology

## Abstract

Podocytopathy, which is characterized by injury to podocytes, frequently causes proteinuria or nephrotic syndrome. There is currently a paucity of effective therapeutic drugs to treat proteinuric kidney disease. Recent research suggests the possibility that glycosphingolipid GM3 maintains podocyte function by acting on various molecules including nephrin, but its mechanism of action remains unknown. Here, various analyses were performed to examine the potential relationship between GM3 and nephrin, and the function of GM3 in podocytes using podocytopathy mice, GM3 synthase gene knockout mice, and nephrin injury cells. Reduced amounts of GM3 and nephrin were observed in podocytopathy mice. Intriguingly, this reduction of GM3 and nephrin, as well as albuminuria, were inhibited by administration of valproic acid. However, when the same experiment was performed using GM3 synthase gene knockout mice, valproic acid administration did not inhibit albuminuria. Equivalent results were obtained in model cells. These findings indicate that GM3 acts with nephrin in a collaborative manner in the cell membrane. Taken together, elevated levels of GM3 stabilize nephrin, which is a key molecule of the slit diaphragm, by enhancing the environment of the cell membrane and preventing albuminuria. This study provides novel insight into new drug discovery, which may offer a new therapy for kidney disease with albuminuria.

## Introduction

Podocyte injury may lead to podocytopathies, represented as either minimal change disease (MCD) or focal segmental glomerulosclerosis (FSGS)^1^. Moreover, podocyte detachment has been documented in many proteinuric diseases and this damage is permanent because podocytes are terminally differentiated. Therefore, it is crucial to preserve the number/quality of podocyte cells to prevent or inhibit the progression of podocytopathies.

Recently, there have been several reports concerning the function of glycosphingolipids in glomeruli^2^ that suggest glycosphingolipid GM3 (Neu5Acα2,3Galβ1,4Glcβ1,1Cer) may be co-localized with nephrin in normal podocytes^3^, and possibly associated with vascular endothelial cell growth factor^4^. In addition, we have found that both MCD and FSGS patients had decreased GM3 and nephrin expression compared with healthy subjects, and in both MCD and FSGS patients, GM3 expression was negatively correlated with proteinuria^5^. However, the relationship between GM3 and glomerular function, especially in terms of podocytes, remains unclear^2^. Glycosphingolipids, which make up part of the cell membrane, are important in transduction and regulation of various extracellular stimuli with intracellular activities^6^. For example, glycosphingolipid GM3 in squamous cell carcinoma and glioblastoma regulates several receptors (e.g. epidermal growth factor receptor, insulin receptor). Interaction of a receptor with GM3 can lead to inhibition of receptor signaling at the cell membrane, resulting in suppression of cellular proliferation^7–10^. Excessive accumulation of glycosphingolipid in the lysosome membrane results in abnormal intracellular signaling^11,12^. Indeed, overexpressed glycosphingolipid on the cell membrane modulates membrane protein function^10,13^.

Several studies have reported on the therapeutic effect of valproic acid (VPA) for albuminuria^14,15^. Moreover, VPA is known as an up-regulator of GM3 synthase gene (*ST3GAL5*; CMP-NeuAc:lactosylceramide α2,3-sialyltransferase; EC2.4.99.9) that increases the level of glycosphingolipid GM3^13,16,17^. Therefore, we reasoned that VPA may help alleviate kidney disease by causing increased expression of GM3. To demonstrate the above, we performed experiments using podocytopathy mice that showed the administration of VPA had a preventive effect on kidney disease. In order to examine the importance of GM3, the same experiment was performed using GM3 synthase gene knockout mice. Furthermore, Watts et al. recently reported that anti-nephrin antibodies are implicated in MCD patients^18^, a disease which at least some degree resembles to the phenotype of anti-Nphs Ab-induced podocytopathy mice in this study.

In this study, we examined whether albuminuria, glomerulosclerosis and podocyte foot process effacement were inhibited by elevated levels of GM3 brought about by administration of VPA using anti-nephrin antibody (anti-Nphs Ab)-induced podocytopathy mice^19^. These findings suggest up-regulation of *ST3GAL5* is effective as a novel therapeutic and preventive strategy for kidney disease with podocytopathy.

## Results

### Expression of nephrin and GM3 in mouse glomeruli

**Figure 1:**
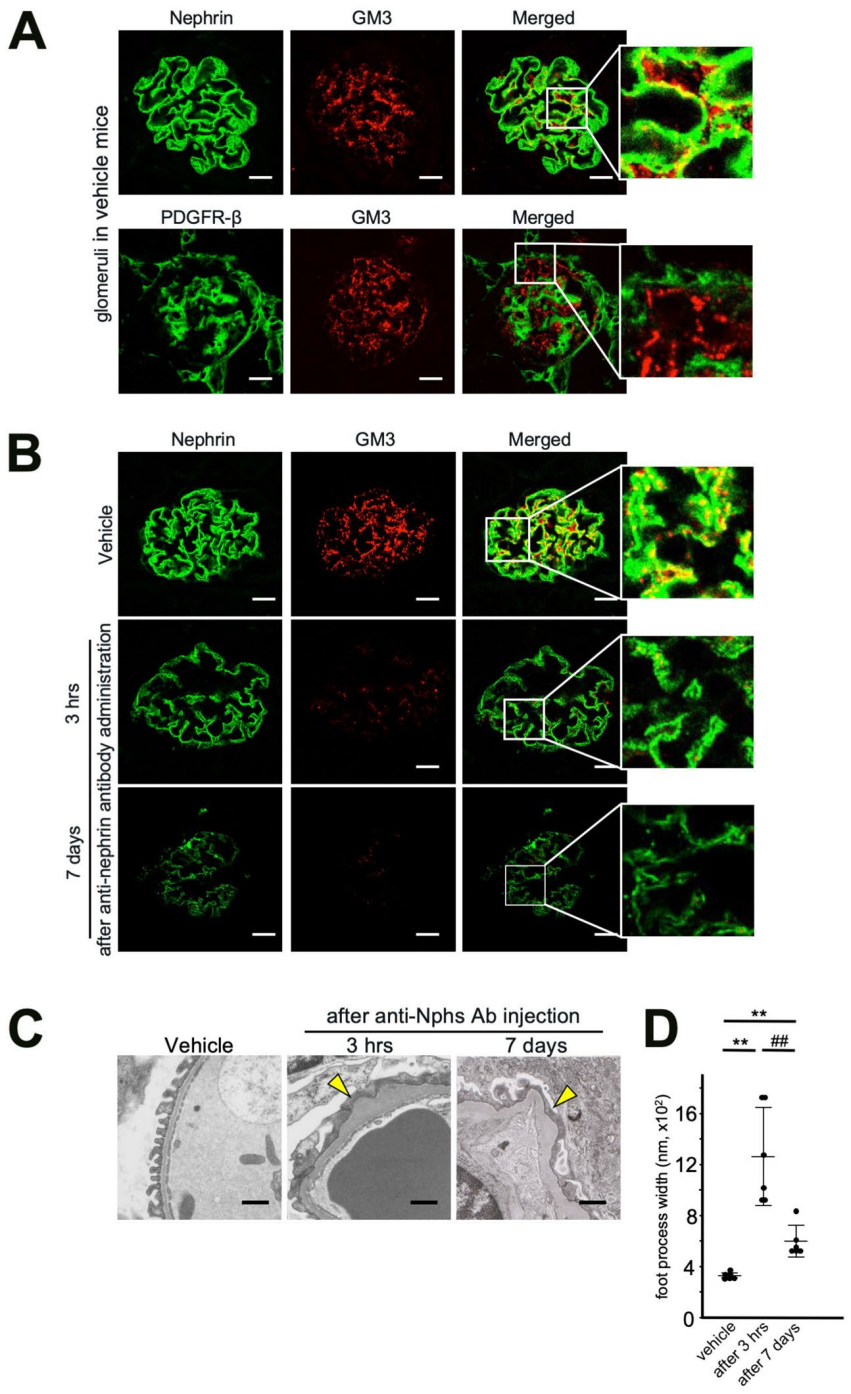
Localization of Nephrin, PDGFR-β and GM3 in glomeruli and the effect of anti-nephrin antibody on the podocyte foot process. (**A**) Immunofluorescence staining images of glomeruli in vehicle mice. Nephrin, or PDGFR-β and GM3 merged florescence images of the glomeruli of vehicle mice. Scale bars: 20 µm. Nephrin (green), or PDGFR-β (green) and GM3 (red) merged areas (yellow) highlighted in enlarged images. (**B**) Immunofluorescence staining images of glomeruli in 1.5 mg of anti-nephrin antibody (anti-Nphs Ab)-induced podocytopathy mice. Scale bars: 20 µm. Nephrin (green) and GM3 (red) merged areas (yellow) highlighted in enlarged images. (**C**) Transmission electron microscopy images of podocyte foot processes in vehicle mice and anti-Nphs Ab-induced podocytopathy mice. Yellow arrowheads showing areas of foot process effacement. Scale bars: 10 µm. (**D**) Scatter diagram showing quantification of foot process effacement. **P < 0.01 vs. vehicle, ^##^*P* < 0.01 vs. 3 h after antibody administration. Statistical analyses were performed from mice (n = 6) in each group.

Our results showed both nephrin and GM3 were expressed in glomeruli of normal mouse kidney (Fig. 1A,B). However, by the 7 day after administration with 1.5 mg of anti-Nphs Ab, levels of nephrin and GM3 were drastically decreased (Fig. 1B). The podocyte foot processes in vehicle mice were normal. By contrast, podocyte foot process effacement (FPE) was evident only 3 hrs after anti-Nphs Ab injection (Fig. 1C,D). These results suggest that GM3 cooperates with nephrin and that this might be related to podocytopathy.

### Preventive effect of podocytopathy by GM3 via administration of VPA

**Figure 2:**
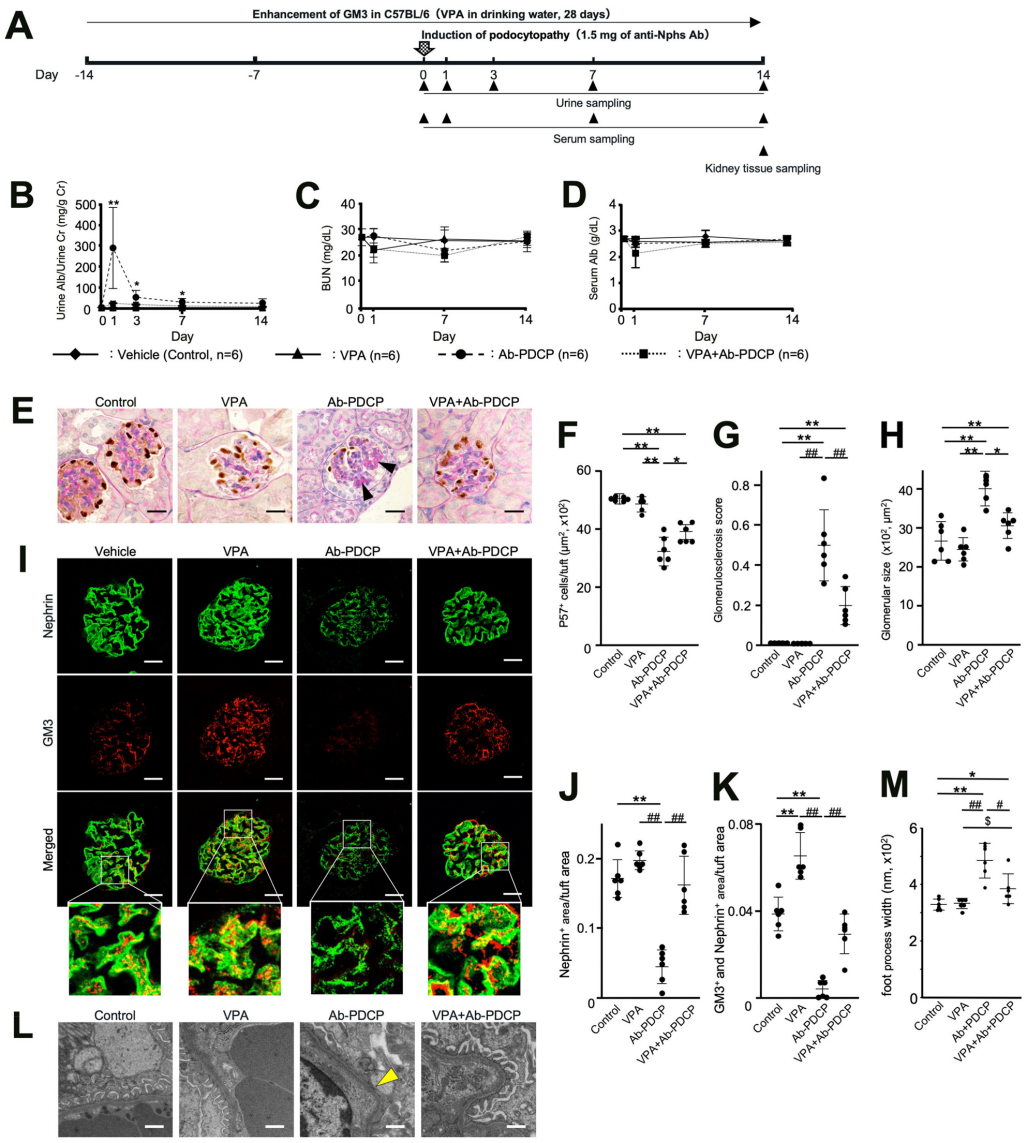
Preventive effect of podocytopathy by GM3 via administration of VPA in anti-nephrin antibody-induced podocytopathy mice. (**A**) Schedule for podocytopathy prevention test using VPA. (**B**) Line graph showing urine albumin to creatinine, (**C**) urea nitrogen (BUN), (**D**) serum albumin. Vehicle (Control), VPA administered mice (VPA), 1.5 mg of anti-nephrin antibody (anti-Nphs Ab)-induced podocytopathy (Ab-PDCP), and VPA + 1.5 mg of anti-Nphs Ab-induced podocytopathy (VPA + Ab-PDCP) mice are shown as a solid line with diamond, a solid line with triangle, dashed line with circle and dotted line with square, respectively. *P < 0.05; **P < 0.01 vs. control. (**E**) p57 (podocyte marker, brown) and periodic acid-Schiff (PAS) staining of glomeruli. Arrowhead showing glomerulosclerosis. Scale bars: 20 µm. (**F**) Scatter diagram showing p57 + cells/tuft, (**G**) glomerulosclerosis score, and (**H**) glomerular size, respectively. Twenty glomeruli per mouse were analyzed in each dot. *P < 0.05; **P < 0.01 vs. control, ^##^*P* < 0.01 vs. Ab-PDCP. (**I**) Immunofluorescence staining images of glomeruli. Scale bars: 20 µm. Nephrin (green) and GM3 (red) merged areas (yellow) highlighted in enlarged images. (**J**) Scatter diagram showing nephrin fluorescence area/tuft area, (**K**) GM3 and nephrin merged fluorescence area/tuft area. **P < 0.01 vs. control, ^##^*P* < 0.01 vs. Ab-PDCP. (**L**) Images of podocyte foot processes by transmission electron microscopy. Yellow arrowhead showing area of foot process effacement. Scale bars: 10 µm. (**M**) Scatter diagram showing quantification of foot process effacement. *P < 0.05; **P < 0.01 vs. control, ^#^*P* < 0.05; ^##^*P* < 0.01 vs. Ab-PDCP, ^$^*P* < 0.05 vs. VPA. Statistical analyses were performed from mice (n = 6) in each group.

We previously reported that VPA can up-regulate and enhance GM3 expression via *ST3GAL5* induction^13,16,17^. Therefore, we reasoned that enhanced GM3 expression induced by VPA might prevent anti-Nphs Ab-induced podocytopathy brought on by administration of 1.5 mg anti-Nphs Ab. To demonstrate whether GM3 can ameliorate podocytopathy, prevention tests with VPA were performed using mice displaying podocytopathy (Fig. 2A). Although there was no significant difference between blood urea nitrogen (BUN) and serum albumin levels, albuminuria levels were clearly different in VPA + podocytopathy mice compared with podocytopathy mice (Fig. 2B–D). The number of p57-positive cells (podocytes) decreased, and glomerulosclerosis increased in podocytopathy mice, compared with control. However, the number of podocytes and glomerulosclerosis were improved in VPA + podocytopathy mice (Fig. 2E–H). Nephrin and GM3 were clearly decreased in the glomeruli of podocytopathy mice, compared with control mice on day 1, 7, 14 after anti-Nphs Ab injection (Fig. 2I–K, Supplementary Fig. S1). Especially, GM3 was barely detectable on day 1 after anti-Nphs Ab injection, when albuminuria was most prevalent (Fig. 2G, Supplementary Fig. S1F). However, levels of nephrin and GM3 in the glomeruli of VPA + podocytopathy mice were almost the same as the control. GM3 expression was more enhanced in VPA administered mice compared with control. Moreover, FPE in podocytopathy mice were significantly blocked by VPA pre-administration (Fig. 2L,M). These results clearly demonstrate that elevated levels of GM3 brought about by administration of VPA had a preventive effect on anti-Nphs Ab-induced podocytopathy.

### Disruption of GM3 synthase gene (*St3gal5*^−/−^) induced podocytopathy

**Figure 3:**
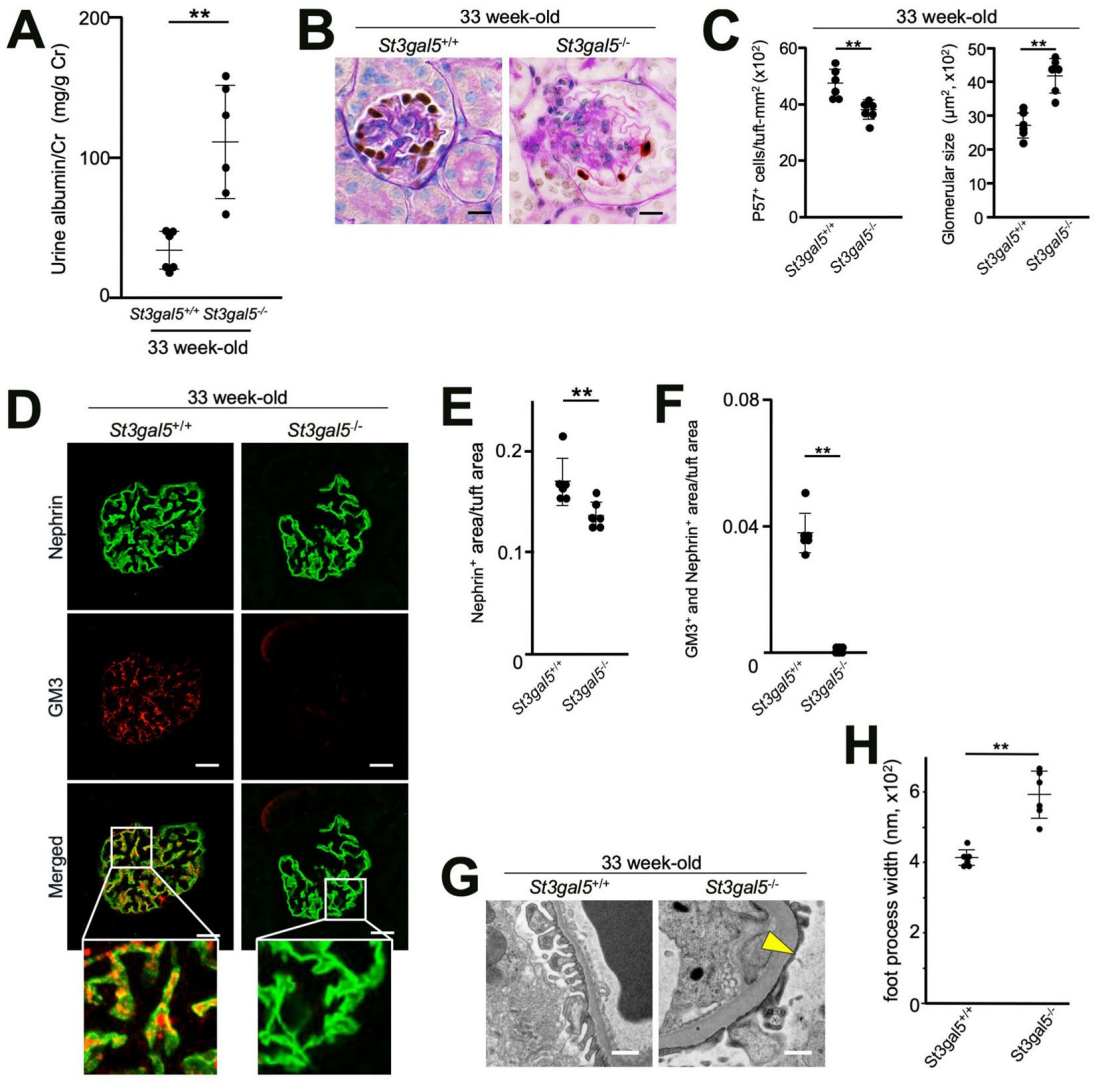
Podocytopathy in aged GM3 synthase gene knockout (*St3gal5*^−/−^*)* mice. (**A**) Scatter diagram showing urine albumin to creatinine. **P < 0.01 vs. wild-type (*St3gal5*^+/+^) mice. (**B**) p57 (podocyte marker, brown) and PAS staining of glomeruli. Scale bars: 20 µm. (**C**) Bar graphs showing p57 + cells/tuft (left) and glomerular size (right). **P < 0.01 vs. *St3gal5*^+/+^. (**D**) Immunofluorescence staining images of glomeruli. Scale bars: 20 µm. Nephrin (green) and GM3 (red) merged areas (yellow) highlighted in enlarged images. (**E**) Scatter diagram showing nephrin fluorescence area/tuft area, (**F**) GM3 and nephrin merged fluorescence area/tuft area. **P < 0.01 vs. *St3gal5*^+/+^. (**G**) Images of podocyte foot processes by transmission electron microscopy. Yellow arrowhead showing areas of foot process effacement. Scale bars: 10 µm. (**H**) Scatter diagram showing quantification of foot process effacement. **P < 0.01 vs. *St3gal5*^+/+^. Twenty glomeruli per mouse were analyzed in each group. Statistical analyses were performed from mice (n = 6) in each group.

**Figure 4:**
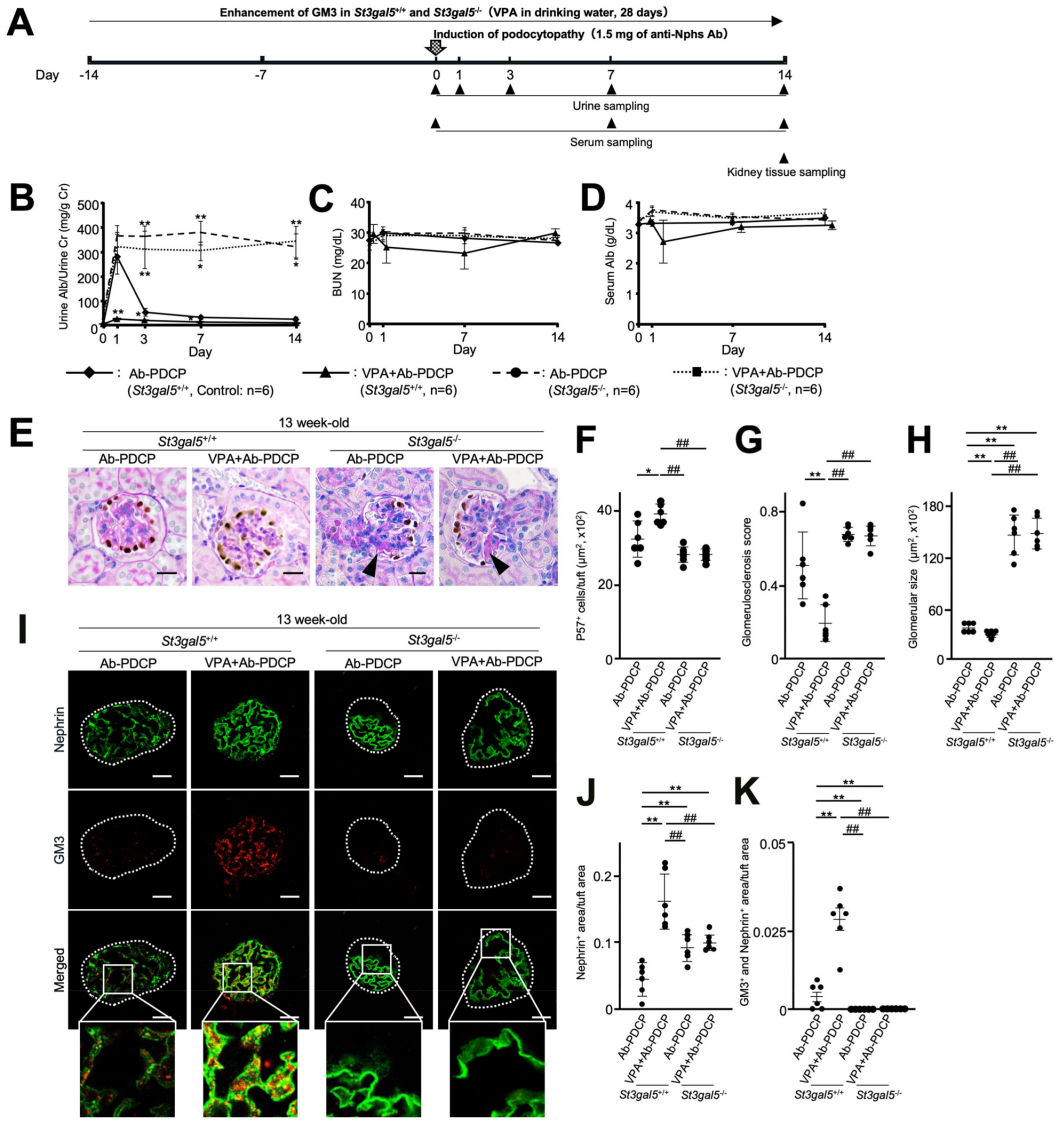
No preventive effect by administration of VPA in young GM3 synthase gene knockout (*St3gal5*^−/−^*)* mice. (**A**) Schedule for podocytopathy prevention test using VPA. (**B**) Line graph showing urine albumin to creatinine, (**C**) urea nitrogen (BUN), (**D**) serum albumin (c). 1.5 mg of anti-nephrin antibody (anti-Nphs Ab)-induced podocytopathy (Ab-PDCP), and VPA + 1.5 mg of anti-Nphs Ab-induced podocytopathy (VPA + Ab-PDCP) in *St3gal5*^+/+^ and *St3gal5*^−/−^ mice are shown as a solid line with diamond, a solid line with triangle, dashed line with circle and dotted line with square, respectively. *P < 0.05; **P < 0.01 vs. *St3gal5*^+/+^. (**E**) p57 (podocyte marker, brown) and PAS staining of glomeruli. Arrowhead showing glomerulosclerosis. Scale bars: 20 µm. (**F**) Scatter diagram showing p57 + cells/tuft, (**G**) glomerulosclerosis score, and (**H**) glomerular size, respectively. Twenty glomeruli per mouse were analyzed in each dot. *P < 0.05; **P < 0.01 vs. Ab-PDCP in *St3gal5*^+/+^, ^##^*P* < 0.01 vs. VPA + Ab-PDCP in *St3gal5*^+/+^. (**I**) Immunofluorescence staining images of glomeruli. Scale bars: 20 µm. Nephrin (green) and GM3 (red) merged areas (yellow) highlighted in enlarged images. (**J**) Scatter diagram showing nephrin (green) fluorescence area/tuft area, (**K**) GM3 (red) and nephrin (green) merged fluorescence area/tuft area. **P < 0.01 vs. Ab-PDCP in *St3gal5*^+/+^, ^##^*P* < 0.01 vs. VPA + Ab-PDCP in *St3gal5*^+/+^. Statistical analyses were performed from mice (n = 6) in each group.

We identified albuminuria that occurred in 33 week-old *St3gal5*^−/−^ mice (Fig. 3A). The glomeruli of *St3gal5*^−/−^ mice had reduced numbers of podocytes (Fig. 3B,C). In 33 week-old *St3gal5*^−/−^ mice, the expression of nephrin also tended to decrease (Fig. 3D–F). Moreover, *St3gal5*^−/−^ mice had glomerular hypertrophy compared with *St3gal5*^+/+^ mice. FPE was observed in the kidneys of 33 week-old *St3gal5*^−/−^ mice (Fig. 3G,H), suggesting these mice showed podocytopathy. Next, prevention tests with VPA were performed using 13 week-old *St3gal5*^−/−^ mice administered with 1.5 mg of anti-Nphs Ab, equivalent to mice shown in Fig. 2A (Fig. 4A). The *St3gal5*^−/−^ mice showed markedly enhanced sensitivity to anti-Nphs Ab injury. Specifically, administration of VPA did not inhibit albuminuria (Fig. 4B–D) and the appearance of podocyte detachment and sclerotic lesions (Fig. 4E–H) in podocytopathy and VPA + podocytopathy *St3gal5*^−/−^ mice. In addition, immunofluorescence staining of 13 week-old *St3gal5*^−/−^ mice clearly showed the level of nephrin was reduced after administration of anti-Nphs Ab. Moreover, even if VPA was pre-administered, nephrin injury caused by anti-Nphs Ab could not be prevented (Fig. 4I–K). These results suggest that the effect of VPA was to act as a histone deacetylase (HDAC) inhibitor except increase of GM3 expression via *ST3GAL5* upregulation did not inhibit podocytopathy. Hence, GM3 is essential for glomerular maintenance (Figs. 2, 3, 4, Supplementary Fig. S1).

### Effect of anti-nephrin antibody and VPA on nephrin injury in model cells

**Figure 5:**
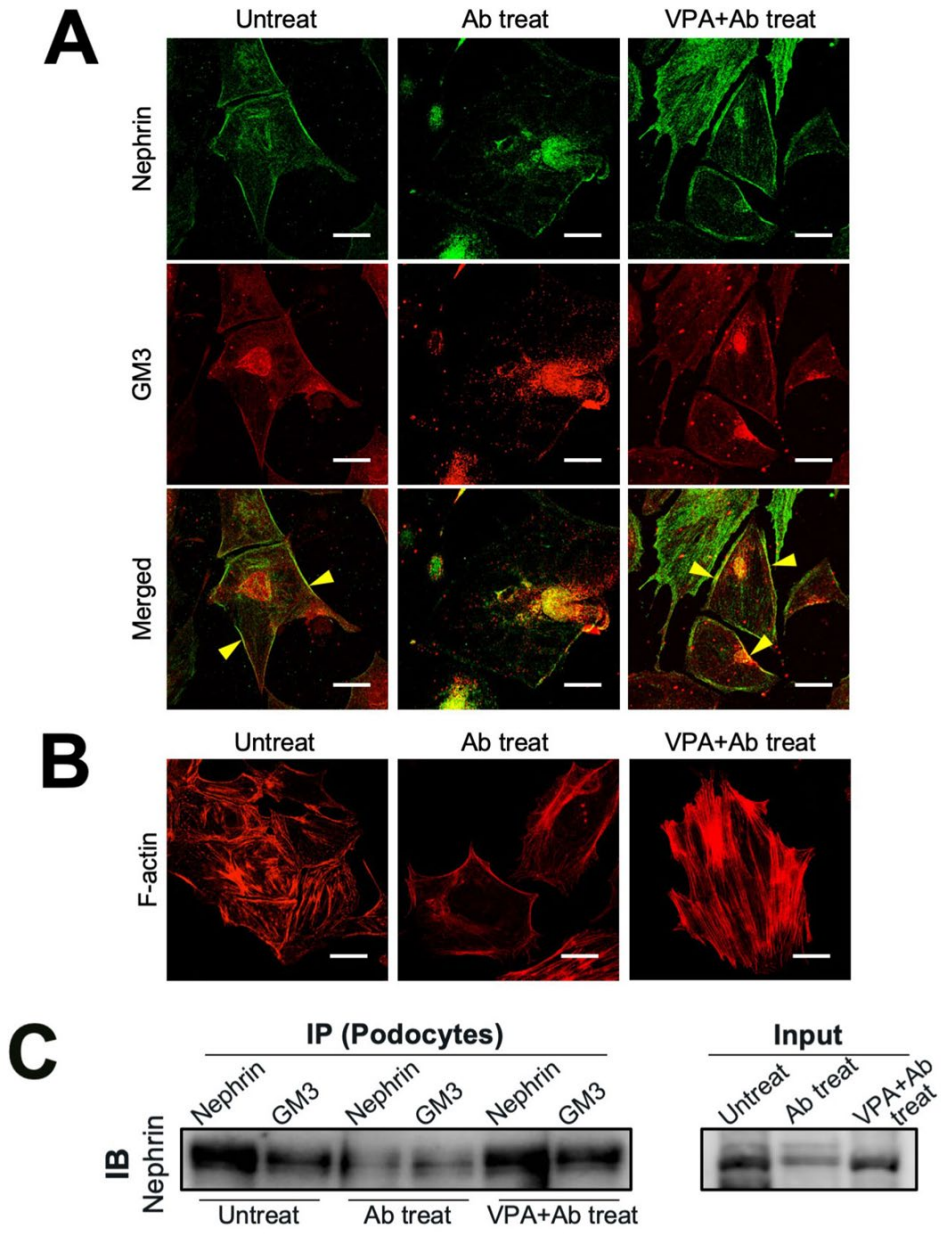
Effect of anti-nephrin antibody and VPA on podocytes and interaction of nephrin and GM3. (**A**) Immunofluorescence staining images of nephrin (green) and GM3 (ref) in various treated podocytes. Yellow arrowheads showing merged areas of nephrin (green) and GM3 (red). (**B**) Immunofluorescence staining images of F-actin (red) in the same treated cells as A. Scale bars: 10 µm. Untreated (Untreat), anti-Nphs Ab treated (Ab treat), preVPA + anti-Nphs Ab treated (VPA + Ab treat) podocytes. (**C**) Immunoprecipitation (IP) using cell lysate of various treated podocytes and anti-nephrin and GM3 antibodies, and immunoblot (IB) analysis using anti-Nphs antibody. Cell lysate of untreated (Untreat), anti-Nphs Ab treated (Ab treat), preVPA + anti-Nphs Ab treated (VPA + Ab treat) podocytes.

To investigate whether nephrin injury could be prevented by elevated levels of GM3 via administration of VPA using podocytes and HEK293/Nephrin (HEK/Nphs) cells. The expression of GM3, nephrin and F-actin bundle assembly as an indicator of healthy cells were compared under various treatment conditions (Fig. 5A,B, Supplementary Fig. S3A). As a result, after anti-Nphs Ab treatment GM3 was barely detectable and the levels of nephrin and F-actin bundle assembly were decreased. However, VPA treatment elicited an increase in the level of nephrin, GM3 and F-actin bundle assembly despite anti-Nphs Ab treatment (Fig. 5A,B, Supplementary Fig. S3A). Immunocytochemistry also indicated a correlation with anti-Nphs Ab-induced podocytopathy mice. Next, we examined VPA specificity for enhancement of GM3 expression in glycosphingolipid biosynthesis via induction of *ST3GAL5*. Exhaustive analysis of the expression of all glycosphingolipid-glycans in HEK/Nphs cells was performed using Glycoblots and Tandem-MALDI-TOF/MS^20–23^ (Supplementary Fig. S4A–C). The molecular species were analyzed and quantified using various treated HEK/Nphs cells. The amount of total ganglio-series of gangliosides (Gg), especially GM3 (Neu5Acα2,3Galβ1,4Glc), in anti-Nphs Ab treated cells decreased compared with untreated cells. By contrast, VPA + anti-Nphs Ab treated cells displayed an increase in the amount of total Gg expression compared to untreated cells. Thus, VPA treatment elicited an increase in the level of total Gg, especially GM3 (Neu5Acα2,3Galβ1,4Glc), even though the cells were treated with anti-Nphs Ab at the same time. There was, however, no significant change in the levels of Gg except for GM3, lacto-series of gangliosides (Lc) and total globo-series of gangliosides (Gb) under the various treatment conditions. Thus, induction of *ST3GAL5* by VPA specifically enhanced GM3 expression and did not affect the level of other glycosphingolipids at the cell membrane as observed from in vivo and in vitro analyses (Figs. 2, 5, Supplementary Table S2, Supplementary Figs. S1–S4).

### Interaction of nephrin and GM3 and their localization in the cell membrane

The immunoprecipitation results using podocytes and HEK/Nphs cells showed GM3 interacted with nephrin in untreated cells (Fig. 5C, Supplementary Fig. S3B). Moreover, the amount of nephrin decreased in anti-Nphs Ab treated cells. By contrast, nephrin levels were maintained in VPA + anti-Nphs Ab treated cells. This finding indicated nephrin interacts with GM3.

We investigated whether escape from nephrin injury by enhanced GM3 expression was due to interaction between nephrin and GM3. Nephrin and Fyn, which were originally localized in raft fractions (fraction 1–3; Glycolipid enriched membrane (GEM)), mostly shifted to non-raft fractions (fraction 4–10; non-GEM) after anti-Nphs Ab treatment (Supplementary Fig. S4D,E). In addition, caveolin-1 (a raft marker) also shifted in the same manner as nephrin. However, nephrin, Fyn and caveolin-1 were restored in the raft fractions by VPA treatment even though the cells were treated with anti-Nphs Ab. As observed with the shift of nephrin, GM3 tended to shift from raft fractions to non-raft fractions upon anti-Nphs Ab treatment (Supplementary Fig. S4F,G). Nonetheless, despite anti-Nphs Ab treatment, GM3 was restored to the raft fractions by VPA treatment to give a distribution akin to that seen for untreated cells. These results indicated that under normal conditions nephrin, GM3, Fyn and caveolin-1 were all localized in raft fractions. However, under abnormal membrane conditions, such as when anti-Nphs Ab interacts with nephrin, raft fractions were disordered and nephrin, GM3, Fyn and caveolin-1 shifted to non-raft fractions. Thus, these experiments also indicated that nephrin was able to escape injury induced by various extracellular factors via enhanced levels of GM3.

## Discussion

The important finding to emerge from this study was that glycosphingolipid GM3 can be used to prevent albuminuria. Of particular importance is the preventive effect observed when GM3 was given to mice with podocytopathy.

We found that GM3 levels decreased together with nephrin in glomeruli of a podocytopathy mouse model induced by administration of anti-Nphs Ab (Fig. 1). Recently, the relationship between GM3 and maintenance of the slit diaphragm in podocytes as well as GM3 and cell adhesion has been reported^4,24^. Here, we demonstrated the preventive effect of GM3 in anti-Nphs Ab-induced podocytopathy mice (Fig. 2) occurs via a newly identified mechanism involving GM3 enhancement induced by VPA^13,16,17^. Specifically, we found that induction of sufficient GM3 in podocyte cell membranes stabilized the slit diaphragm components, including nephrin (Fig. 5). The overall effect of GM3 induction was to inhibit podocyte detachment (Figs. 2, 3, 4, 5, Supplementary Figs. S1–S4). Moreover, we performed VPA preventive tests of podocytopathy induced by administration of anti-Nphs Ab using *St3gal5*^−/−^ mice (Fig. 4). Originally, *St3gal5*^−/−^ mice were established to investigate the relationship between GM3 and insulin resistance^25^*.* Here, for the first time, we focused on the effect of GM3 on kidney (Figs. 3, 4). From our examinations, the following results were established. In young *St3gal5*^−/−^ mice (13 week-old) there were no significant differences in nephrin expression by comparison to wild-type mice (Fig. 3). Nonetheless, *St3gal5*^*-/—*^mice showed podocytopathy with irreversible albuminuria by anti-Nphs Ab administration.

Furthermore, in young *St3gal5*^−/−^ mice (13 week-old), there were only very minor changes in the glomeruli (Supplementary Fig. S2). However, the loss of podocytes is not reflected in obvious symptoms. Thus, kidney function of these mice is maintained by components other than glycolipids (cholesterol, tetraspanin etc.)^26^. Nonetheless, once positively stimulated by anti-Nphs Ab, there is a transition to an irreversible and severe disorder. It should be noted that proteinuria and sclerotic lesions appear even under unstimulated conditions in 33 week-old mice, unlike young mice. This effect presumably arises from factors associated with aging. It is likely that in the absence of GM3 other molecules participate in incomplete raft formation to maintain kidney function in *St3gal5*^*-/—*^mice. In this study, using *St3gal5*^−/−^ mice, we found that incomplete rafts cannot recover from albuminuria induced by an anti-Nphs Ab. Thus, taking these observations into consideration, anti-Nphs Ab-inducing podocytopathy is a disorder caused by an imbalance of glycosphingolipids in the cells. The expression of GM3 is crucial for maintaining a healthy kidney.

Our results showed that nephrin, GM3 and Fyn, which were originally localized in the raft fraction, shift to the non-raft fraction by anti-Nphs Ab treatment (Supplementary Fig. S4D,E). Fyn is a tyrosine kinase involved in a variety of functions including regulation of survival^27^, cell adhesion^28^, integrin mediated signaling^29–31^ and cytoskeletal remodeling^32–35^. There are many reports focusing on the relationship between nephrin and cytoskeletal remodeling^36–38^. However, our results indicated that abundant levels of GM3 in the raft fraction can inhibit the shift of nephrin and Fyn to the non-raft fraction even during anti-Nphs Ab treatment. Thus, nephrin injury caused by anti-Nphs Ab was reduced by enhanced GM3 expression in the cell membrane. This observation may indicate the mechanism by which GM3 elicits a preventive effect on albuminuria in anti-Nphs Ab-induced podocytopathy mice.

Jin et al. reported that the soluble form of vascular endothelial growth factor (VEGF) receptor (sFlt1), which is expressed on podocytes, binds GM3 enriched lipid-rafts in the cell membrane of podocyte foot processes, thereby controlling cytoskeleton reconstitution and cell adhesion via activation of nephrin and syndecan protein family members^4^. Furthermore, sFlt1 and GM3 might have a cooperative role in maintaining normal kidney function. Indeed, VEGF controls and assembles various protein functions on lipid-rafts including nephrin phosphorylation^37,38^, cytoskeleton reconstitution^36^ and adhesion in podocytes^38,39^. However, except for one study suggesting GM3 is required for normal kidney function in human, which was based on immunoelectron microscopy^3^, there have been no further reports on this matter.

There are several reports focusing on the therapeutic effect of VPA on albuminuria. Studies on adriamycin-induced nephropathy mice, lipopolysaccharide mice and diabetic nephropathy rats^14,15^ and one involving a cohort of US veterans^15^ reported that VPA was particularly effective at inhibiting the progression of renal dysfunction in a group of patients with severe albuminuria. VPA acts not only as an up-regulator of *ST3GAL5* (or *St3gal5*) but also as an HDAC inhibitor. The therapeutic mechanism in these studies was presumed to involve the anti-inflammatory effect mediated by the HDAC class I inhibitory action of VPA. Moreover, other researchers have found the therapeutic effect of Trichostatin A, which exhibits HDAC class I inhibition, was more potent than VPA, although weaker in adriamycin-induced nephropathy mice^40–42^. In this study, our observations demonstrated VPA was ineffective in preventing podocytopathy in *St3gal5*^*-/—*^mice. These results clearly showed that GM3 is essential for glomerular maintenance of normal mouse kidney. Therefore, we suggest that the primary biological action of VPA related to these observations in preventing albuminuria is elevated levels of GM3 via up-regulation of *ST3GAL5*, which might be related to its HDAC inhibitory activity. Moreover, this proposal is strongly supported by our observation of anti-Nphs Ab-induced podocytopathy in *St3gal5*^−/−^ mice despite pre-administration of VPA.

This study has demonstrated the preventive mechanism mediated by GM3 using several animal and cell models. Our findings suggest glycosphingolipids, especially GM3, play a fundamental role in maintaining podocyte function (slit diaphragm stabilization, cell survival, etc.). We propose that the inhibition mechanism of albuminuria occurs via enhancement of GM3, which localizes around the nephrin transmembrane domain to inhibit changes in the conformation of nephrin caused by anti-Nphs Ab binding. Hence, the overall effect of the preventive treatment is to maintain nephrin on the cell surface, retaining its function and the integrity of the slit diaphragm, thereby sustaining the glomerular filtration barrier. These findings suggest a new therapeutic approach for podocytopathy.

## Methods

See Supplementary Information for additional information.

### Animal study

C57BL/6N mice (male, 7 week-old, 19–20 g) were purchased from CLEA Japan Inc. (Tokyo, Japan) and GM3 synthase gene knockout (*St3gal5*^−/−^) mice were generated by Dr. Yamashita et al*.* as described previously^25^. C57BL/6 N mice and GM3 synthase gene knockout (*St3gal5*^−/−^) mice (male, 13 and 33 week-old, 20–25 and 27–30 g) were housed in metabolic cages under specific-pathogen-free conditions and fed a standard chow.

We previously reported an anti-mouse nephrin polyclonal antibody (anti-Nphs Ab) raised in rabbits and generated using a genetic immunization method^19^. Antibody from antisera was purified using nProtein A Sepharose Fast Flow (#17528001; GE Healthcare, Chicago, IL, USA). For in vivo testing, mice were first randomized into three groups: i) control group, ii) VPA treated group, (iii) anti-Nphs Ab-induced podocytopathy group, iv) VPA treated anti-Nphs Ab-induced podocytopathy group (each n = 6, respectively). To induce podocytopathy in mice, C57BL/6 and *St3gal5*^−/−^ mice were injected with a single aliquot of either 1.5 mg of anti-Nphs Ab via the tail vein. VPA was administrated as 4 mM VPA in drinking water (100 mg/kg/day as human equivalent dose). This dosage is ensured safety as drugs in human)^43^. Kidney tissues from three groups of C57BL/6 mice (groups of on day 1, 7, 14 after anti-Nphs Ab administration) were sampled and used for various analyses.

### Tissue staining

Kidney tissues with p57 and periodic acid-Schiff (PAS) staining was assessed as previously described^44,45^. Glomerulosclerosis was graded quantitatively by the percentage of glomerular tuft area involvement as follows^19^: score 0, no sclerosis; score 1, < 25%; score 2, 25–50%; score 3 50–75%; score 4, 75–100%. Global sclerosis means glomeruli with more than 75% tuft area scleroses, which is a score of 4. Immunofluorescence staining of nephrin and GM3 were carried out using frozen sections of mouse kidney tissues with various antibodies. Pathological images were visualized by optical microscopy (BX51; Olympus, Tokyo, Japan) and analyzed by ImageJ software (https://imagej.nih.gov/ij/). Fluorescence images were obtained using a confocal laser-microscope (LSM710; Carl Zeiss, Oberkochen, Germany) and analyzed by ZEN imaging software (Carl Zeiss).

### Glycosphingolipid-glycan preparation by glycoblotting method

Approximately 1 × 10^6^ cells were suspended in 200 µL of PBS and homogenized using an Ultrasonic Homogenizer. Ethanol precipitation was carried out by incubation at − 30 °C for 16 h. The cellular proteinaceous pellet and supernatant/lipid fractions were separated by centrifugation. Cellular pellets were dissolved in 100 µL of water and the protein concentration was measured using BCA protein assay kit (#23227; Thermo Fisher Scientific, Waltham, MA, USA). To release intact glycosphingolipid-glycans, the supernatant/lipid fractions corresponding to 400 µg protein were dried to remove ethanol and then resuspended in 45 µL of 50 mM acetate buffer, pH 5.5, containing 0.2% Triton X-100. Enzymatic digestion was performed by the addition of 5 µL endoglycoceramidase I (EGCase I) followed by incubation at 37 °C for 16 h^23^. To take account of free oligosaccharide (fOSs) contamination in the lipid fractions, non-enzymatic digestion samples were prepared as negative controls^21^. The kidney tissues were homogenized in 100 µL PBS(-) using an beads crusher (TAITEC, Saitama, Japan). The method for protein quantification and intact glycosphingolipid-glycan release were the same as procedure described above.

### Glycosphingolipid-glycan purification for MALDI-TOF MS analysis

Released glycosphingolipid-glycans were subjected to a glycoblotting procedure as previously described^20^. In brief, EGCase digested samples (25 µL), using involving an internal standard of Neu5Ac2Gal2GlcNAc2 + Man3GlcNAc1 (A2GN1, 10 pmol), were captured on BlotGlyco® beads (5 mg; Sumitomo Bakelite Co., Ltd, Tokyo, Japan). Unreacted hydrazide groups on beads were capped with acetyl groups by treatment with 10% acetic anhydride in methanol. To protect carboxy groups of sialic acids, methyl esterification was performed with 100 mM 3-methyl-1-*p*-tolyltriazene in dioxane on a solid-phase. Next, these methyl esterified glycans were released and labeled with aminooxy-functionalized tryptophanylarginine methyl ester (aoWR) via a transamination reaction. Excessive aoWR reagent was removed by solid-phase extraction using a HILIC µElution plate (#186002780; Waters, Milford, MA, USA). Purified glycan solutions were mixed with 2,5-dihydroxybenzoic acid (10 mg/mL in 30% ACN) and subsequently subjected to MALDI-TOF MS analysis as previously described^22^. Briefly, all measurements were performed using an Ultraflex II TOF/TOF mass spectrometer equipped with a reflector and controlled by the FlexControl 3.0 software package (Bruker Daltonics, Bremen, Germany) according to general protocols. All spectra were obtained in reflectron mode with an acceleration voltage of 25 kV, a reflector voltage of 26.3 kV, and a pulsed ion extraction of 160 ns in positive ion mode. Masses were annotated using the FlexAnalysis 3.0 software package. SphinGOMAP (http://www.sphingomap.org/) online databases were used for structural identification of GSL-glycans. Absolute quantification was performed by comparative analyses between the areas of the MS signals derived from each GSL-glycan and a known amount of the internal standard (A2GN1).

### Glycosphingolipid extraction and high-performance TLC analysis

Glycosphingolipid extraction was performed as described previously^46,47^. In brief, 2 × 10^8^ cells were harvested using a rubber scraper and washed with PBS. Gangliosides from cells were extracted by sonication 4 times in isopropanol/hexane/water (IP/H/W) (55:25:20, by volume). Extracts were combined, evaporated and dissolved in 6 volumes of chloroform/methanol (CM) (2:1, by volume) and water was added to achieve chloroform/methanol/water (CMW) (4:2:1, by volume). After shaking, the mixture was allowed to separate into upper and lower phases. The upper phases containing glycosphingolipid, which partitioned according to the Folch procedure, were combined and evaporated. Dried upper phases were solubilized in distilled water and the resultant solution was applied to a Sep-Pak Plus C18 cartridge (#WAT020515; Waters, Milford, MA, USA) for desalting. Purified glycosphingolipids were developed on high-performance TLC (HPTLC) plates (#1.05641.0001; Merck, Darmstadt, Germany) with a solvent system consisting of chloroform/methanol/0.2% aqueous CaCl_2_ (CMW) (55:45:10, by volume), and visualized by spraying with 0.5% orcinol in 1 M sulfuric acid.

### Statistical analysis

Data were analyzed as the mean ± SEM. Statistical comparisons between two groups were evaluated by Mann–Whitney’s U-test. Multiple-group comparisons were evaluated using 1-way ANOVA followed by Tukey’s test. Statistical analysis was performed by StatFlex Ver.7 (Artech, Osaka, Japan). A *P*-value of less than 0.05 was considered statistically significant.

### Study approval

All animal experiments were approved by the Ethical Review Committee for Animal Experiments of Kitasato University School of Medicine (#2017-182, #2018-006, #2019-072, #2020-016) and Azabu University of Veterinary Medicine (#201204-2). DNA studies were approved by the Ethical Review Committee for Recombinant DNA Experiments of Kitasato University School of Medicine (#3837). All experiments were performed in accordance with relevant guidelines and regulations and the study is reported in accordance with ARRIVE guidelines.

## Supplementary Information

Supplementary Information.

## Data Availability

The data generated during this study are available on reasonable request.
