# Supplementary material for: Glycosphingolipid GM3 prevents albuminuria and podocytopathy induced by anti-nephrin antibody
Source: Sci Rep. 2022 Sep 26;12:16058. doi: 10.1038/s41598-022-20265-w (PMC9513075; doi:10.1038/s41598-022-20265-w)
Supplement: Supplementary file 1 — Supplementary Information. [file 41598_2022_20265_MOESM1_ESM.pdf]

Supplementary Information

Glycosphingolipid GM3 prevents albuminuria and podocytopathy induced by anti-nephrin antibody

Authors

Nagako Kawashima, Shokichi Naito, Hisatoshi Hanamatsu, Masaki Nagane, Yasuo Takeuchi,

Jun-ichi Furukawa, Norimasa Iwasaki, Tadashi Yamashita and Ken-ichi Nakayama

This file included:

Supplementary Table S1-S2

Supplementary Methods S1-S7

Supplementary Figures and Legends S1-S3

Supplementary Results

References

Supplementary Tables

Supplementary Table S1 Reagent, Antibodies for immunofluorescence, immunocytochemistry labeling, Western blot analysis and immunoprecipitation

**Name**	**Source**	**Type**
Valproic acid sodium salt	FUJIFILM Wako Chemicals, #193-18352	powder
Anti-Nephrin antibody	Original [1] (used for induction of podocytopathy *in vivo* tests)	Mouse, polyclonal
Anti-Nephrin C-terminus antibody	Original (established in this study, used for detection of nephrin *in vivo* tests and *in vitro* experiments)	Mouse, polyclonal
Anti-p57 antibody (H-91)	Santa Cruz Biotech, #sc-8298	Rabbit, polyclonal
Anti-GM3 antibody (GMR6)	Tokyo Chemical Industry, #A2582 (used for detection of GM3 *in vivo* tests and *in vitro* experiments)	Mouse, monoclonal
Anti-PDGFR-β (28E1)	Cell Signaling Tech, #3169	Rabbit, monoclonal

Alexa Fluor555-Phalloidin	Thermo Fisher Scientific, #A34055	-
Anti-Fyn	Cell Signaling Tech, #4023	Human, polyclonal
Anti-Caveolin-1	Cell Signaling Tech, #3238	Human, polyclonal
Goat anti-Rabbit IgG-HRP	Santa Cruz Biotech, #sc-2004	Goat, polyclonal
Alexa488-Rabbit anti-Mouse IgG	Thermo Fisher Scientific, #A27023	Rabbit, polyclonal
Alexa568-Goat anti-Mouse IgG	Thermo Fisher Scientific, #A11004	Rabbit, polyclonal
Alexa Fluor 488-Goat anti-Mouse IgM	Life Technologies, #A-21042	Goat, polyclonal
Alexa Fluor 568-Goat anti-Mouse IgM	Life Technologies, #A-21043	Goat, polyclonal

Supplementary Table S2 Structure analysis of glycosphingolipids

m/z	class	glycosphingolipid name	pmol/ 100 ∝g Protein				structure composition
			Untreated	VPA	Ab	VPA+Ab	
772.34	Lac	LacCer	270.44 ± 95.83	355.23 ± 27.99	192.28 ± 47.11	311.54 ± 72.93	Galβ1,4Glc
934.39	Gb	Gb3	89.56 ± 12.58	173.31 ± 22.14	26.49 ± 14.76	372.37 ± 44.58	Galα1,4Galβ1,4Glc
1045.43	Gg	GM3_lactone	16.45 ± 1.67	23.06 ± 4.27	11.46 ± 2.17	24.65 ± 2.48	Neu5Ac(lac)α2,3Galβ1,4Glc
1077.45	Gg	GM3	1977.69 ± 55.64	2410.34 ± 117.10	1426.74 ± 150.54	2912.98 ± 327.44	Neu5Acα2,3Galβ1,4Glc
1093.45	Gg	GM3(Neu5Gc)	15.46 ± 1.87	22.22 ± 3.15	9.99 ± 1.95	20.49 ± 3.59	Neu5Gcα2,3Galβ1,4Glc
1280.53	Gg	GM2	50.78 ± 7.88	136.52 ± 12.47	62.42 ± 8.47	493.24 ± 40.76	GalNAcβ1,4(Neu5Acα2,3)Galβ1,4Glc
1296.53	Gg	GM2(Neu5Gc)	1.50 ± 0.39	5.74 ± 0.99	1.92 ± 0.45	22.48 ± 4.58	GalNAcβ1,4(Neu5Gcα2,3)Galβ1,4Glc
1410.56	Gg	GM1_lactone	2.33 ± 0.72	3.53 ± 0.41	2.72 ± 0.56	6.14 ± 0.95	Galβ1,3GalNAcβ1,4(Neu5Ac(lac)α2,3)Galβ1,4Glc
1442.58	Gg	GM1	42.49 ± 8.59	51.30 ± 6.02	55.43 ± 7.62	130.24 ± 20.32	Galβ1,3GalNAcβ1,4(Neu5Acα2,3)Galβ1,4Glc
1458.58	Gg	GM1(Neu5Gc)	0.68 ± 0.24	0.96 ± 0.18	1.10 ± 0.27	2.66 ± 0.74	Galβ1,3GalNAcβ1,4(Neu5Gcα2,3)Galβ1,4Glc
1715.67	Gg	GD1a_lactone	0.07 ± 0.13	0.26 ± 0.22	0.66 ± 0.22	3.42 ± 1.09	Neu5Acα2,3Galβ1,3GalNAcβ1,4(Neu5Ac(lac)α2,3)Galβ1,4Glc
1747.70	Gg	GD1a	4.00 ± 1.98	6.21 ± 1.03	12.34 ± 3.25	51.42 ± 13.69	Neu5Acα2,3Galβ1,3GalNAcβ1,4(Neu5Acα2,3)Galβ1,4Glc
975.42	Gg/(n)Lc	asialo-GM2/Lc3	14.80 ± 0.88	22.09 ± 4.30	9.22 ± 2.24	45.71 ± 3.28	GalNAcβ1,4Galβ1,4Glc<br>GlcNAcβ1,3Galβ1,4Glc
1775.69	Gg/(n)Lc	Sialyl-nLc6_lactone	0.16 ± 0.17	0.11 ± 0.19	0.51 ± 0.10	0.00 ± 0.00	Neu5Ac(lac)α2,3Galβ1,3(4)GlcNAcβ1,3Galβ1,3(4)GlcNAcβ1,3Galβ1,4Glc
1807.72	Gg/(n)Lc	GalGalNAc-GM1b/Sialyl-nLc6	7.39 ± 2.57	4.33 ± 0.50	11.45 ± 2.42	1.56 ± 0.35	Galβ1,3GalNAcβ1,4(NeuNAcα2,3)Galβ1,3GalNAcβ1,4Galβ1,4Glc<br>Neu5Acα2,3Galβ1,4GlcNAcβ1,3Galβ1,4GlcNAcβ1,3Galβ1,4Glc
1137.47	Gg/(n)Lc/Gb	asialoGM1/(n)Lc4/Gb4	66.93 ± 0.41	78.59 ± 6.95	51.76 ± 9.90	132.69 ± 5.17	Galβ1,3GalNAcβ1,4Galβ1,4Glc<br>Galβ1,3(4)GlcNAcβ1,3Galβ1,4Glc<br>GalNAcβ1,3 Galα1,4Galβ1,4Glc
1299.52	Gg/(n)Lc/Gb	Gb5(SSEA-3)	1.17 ± 0.26	2.20 ± 0.67	1.19 ± 0.45	3.26 ± 0.16	Galβ1,3GalNAcβ1,3 Galα1,4Galβ1,4Glc
1340.55	Gg/(n)Lc/Gb	asialo-GalNAc-GM1/nLc5	0.63 ± 0.16	1.27 ± 0.31	0.52 ± 0.16	1.87 ± 0.20	GalNAcβ1,4Galβ1,3GalNAcβ1,4Galβ1,4Glc<br>GlcNAcβ1,3Galβ1,4GlcNAcβ1,3Galβ1,4Glc
1502.60	Gg/(n)Lc/Gb	asialo-GalGalNAc-GM1/nLc6/GlcNAc-Gb5	3.73 ± 0.90	6.29 ± 1.80	4.26 ± 0.98	1.79 ± 0.21	Galβ1,3GalNAcβ1,4Galβ1,3GalNAcβ1,4Galβ1,4Glc<br>Galβ1,4GlcNAcβ1,3Galβ1,4GlcNAcβ1,3Galβ1,4Glc<br>GlcNAcβ1,6(Galβ1,3)GalNAcβ1,3 Galα1,4Galβ1,4Glc

Supplementary Methods

Supplementary Method S1 Reagents and Antibodies

Reagents and antibodies are listed in Supplementary Table S1. Anti-mouse nephrin polyclonal antibody (anti-cNphs Ab) was raised against the C-terminus of nephrin in rabbits using synthetic peptides conjugated to keyhole limpet hemocyanin. The sequence of the peptide antigen corresponded to the C-terminal region of mouse nephrin; GEPGSLPFELRGHLVC(C). Antibodies from antisera were purified using peptide antigen conjugated to Sepharose 6B. The anti-cNphs Ab was further purified by passage through nephrin C-terminus peptide conjugated to Sepharose 6B for removal of cross-reactive antibodies.

Supplementary Method S2 Biochemical studies

For podocytopathy preventive tests, urine and blood were sampled at each of the following days: 0, 1, 3, 7, 14 after anti-nephrin antibody (anti-Nphs Ab) injection. Each serum was prepared from blood by centrifugation at 1,200 x g for 20 min at 4°C. Urine albumin, creatinine and nitrogen, serum creatinine and albumin were measured by SRL Co., Ltd.

Supplementary Method S3 Electron microscopy

Mouse kidney cortex samples were fixed in 0.1 M sodium cacodylate buffer (pH 7.4) containing 2.5% glutaraldehyde for 3 hrs at 4°C. After washing in 0.1 M sodium cacodylate buffer (pH 7.4) the samples were fixed in 0.1 M sodium cacodylate buffer (pH 7.4) containing 2% OsO_4 for 1 hr. Following dehydration with ethanol, the buffer was replaced with *n*-butyl glycidyl ether (QY-1; Nisshin EM, Tokyo, Japan) and the samples embedded in epoxy resin (Quetol 812; Nisshin EM). Ultrathin sections (80 nm-thick) of renal cortex were prepared and stained with 3% uranyl acetate and lead citrate. Samples were analyzed using a transmission electron microscope (H-7650; Hitachi, Tokyo, Japan) fitted with a CCD camera (#Veleta; Olympus, Tokyo, Japan).

Supplementary Method S4 Cell establishment and treatment

Mouse podocytes (heat-sensitive mouse podocyte: HSMP) were provided by Dr. Shankland (University of Washington) [2], and human embryonic kidney 293 (HEK293) were purchased from RIKEN BioResource Research Center (Tsukuba, Ibaraki, Japan). All cultured cells were maintained

in basal medium (RPMI 1640 (#36264-85; Nacalai Tesque, Kyoto, Japan), 100 mM HEPES (#17557-94; Nacalai Tesque), penicillin (100,000 units/mL)-streptomycin (10,000 μg/mL) (#09367-34; Nacalai Tesque), 1 mM sodium pyruvate (#06977-34; Nacalai Tesque) containing 10% FBS (#35015121; Corning Inc., New York, NY, USA). HSMP were maintained in RPMI 1640 basal medium supplemented with 50 units/mL mIFN-γ (#11276905001, Sigma-Aldrich) at 33°C in the growth phase and the same medium without mIFN-γ at 37°C in the differentiation phase [2]. HEK293 cells were stably transfected with mouse nephrin gene (*Nphs1*, NM_019459) using FuGENE6 (#E2691; Promega, Madison, WI, USA) transfection reagent and selected by G418 (50 μg/ml, #11811098; Invitrogen, Waltham, MA, USA) (HEK/Nphs). *ST3GAL5* knockout HEK/Nphs cells were established using Edit-R CRISPR-Cas9 genome editing system (GE Healthcare, Chicago, IL, USA) (HEK/Nphs+*ST3GAL5*-KO) and Edit-R hCMV-PuroR-Cas9 nuclease expression plasmid (#U-005100-120; GE Healthcare) were transfected using DharmaFECT Duo Transfection Reagent (#T-2010-01; GE Healthcare) following manufacturing instructions. To obtain the HEK/Nphs-*ST3GAL5* clone, HEK/Nphs cells were transiently transfected with human GM3 synthase gene (*ST3GAL5*: NM_006927) inserted into pcDNA3.1/Zeo(+) plasmid (donated by Dr.

Inokuchi and Dr. Uemura, Tohoku Medical and Pharmaceutical University)[3] (HEK/Nphs+*ST3GAL5*). To obtain nephrin injury cells, anti-Nphs Ab was added to each cell lines and incubate for 24 hrs.

Supplementary Method S5 Immunocytochemistry

Seeded cells onto a glass bottomed dish (#D11130H, Matsunami, Osaka, Japan) were cultured for a few days (each HEK/Nphs cell line) or for 12 days (mouse podocytes). After washing with PBS, cells were fixed with 2% formaldehyde-4% sucrose for 10 min at rt, then washed with PBS, and permeabilized with 0.3% Triton X-100 for 10 min at rt. After blocking, cells were incubated for 16 hrs at 4°C with a primary antibody. Cells were subsequently incubated with a secondary antibody conjugated to a fluorescent dye for 1 hr at 4°C. After washing with PBS, cells were mounted using Fluoromount (#K024; Diagnostic BioSystems, Pleasanton, CA, USA). Fluorescence images were visualized and analyzed using a confocal laser-microscope and software described in Method (Tissue staining) and Photoshop CS4 software (Adobe Systems, San Jose, CA, USA).

Supplementary Method S6 Protein sample preparation and Western blotting

For preparation of cell lysate and cell membrane fractions, cells were cultured to semiconfluent monolayers. Subsequently, 1% SDS-lysis buffer (20 mM HEPES, 1 mM Na_3VO_4, 150 mM NaCl, 1 mM phenylmethylsulfonyl fluoride, 1% aprotinin and protease inhibitors) was added to the cells, which were then lysed for 30 min at 4°C (cell lysate). Lysed cells were separated into fractions by sucrose density-gradient centrifugation as follows. In brief, cells were washed with PBS and suspended in 2 mL TNE buffer (25 mM Tris-HCl (pH 7.5), 150 mM NaCl, 1 mM EDTA containing protease inhibitors and 1 mM Na_3VO_4). Suspended cells were centrifuged for 5 min at 1,300 x g, and the precipitates were resuspended in 2 mL TNE buffer containing 1% TritonX-100, 100 µM PMSF and homogenized with a Dounce-homogenizer. After centrifugation for 5 min at 1,300 x g, the supernatants were diluted with equal volumes of 85% (w/v) sucrose in TNE buffer. In an ultracentrifuge tube the diluted lysates were carefully overlaid with 4 mL 30% (w/v) sucrose in TNE buffer, then with 4 mL 5% (w/v) sucrose in TNE buffer. The samples were centrifuged at 20,000 x g for 18 hrs in an SW41 rotor (Beckman Instruments, Palo Alto, CA, USA) (cell membrane fractions), and 1 mL fractions were collected from the top for Western blotting [4] and TLC analysis [5] described in Method (Glycosphingolipid extraction and High-Performance TLC analysis).

Supplementary Method S7 Immunoprecipitation

Cell pellets obtained from ≈10^6 cells (cultured in 6 cm diameter dishes) were suspended in 300 µL of lysis buffer (1% Triton X-100, 20 mM Tris-HCl (pH 7.4), 150 mM NaCl, 1 mM Na_3VO_4, 1 mM EDTA, 1 mM PMSF, 1% aprotinin and protease inhibitors) and resuspended cells were lysed by incubating for 20 min at 4°C. Supernatants were collected after centrifugation at 17,400 x g for 5 min. Aliquots (200 µg protein) of supernatants were used for immunoprecipitation with nephrin and GM3 antibodies and the resultant precipitates were analyzed by SDS-PAGE followed by Western blotting analysis.

Supplementary Figures and Legends

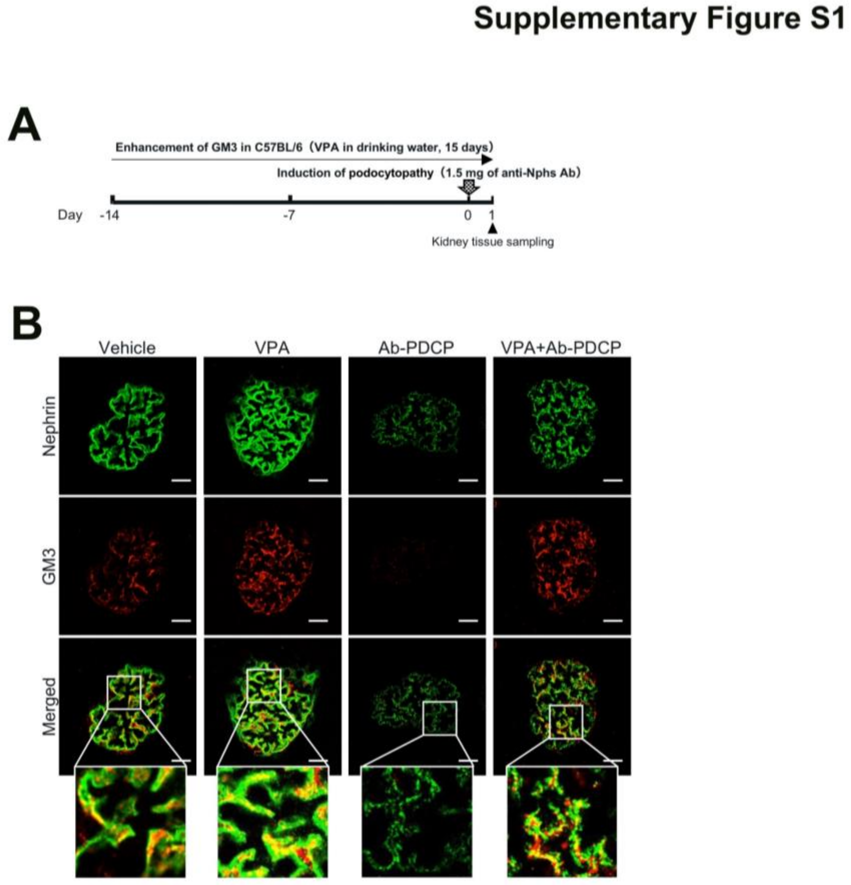

Supplementary Fig. S1. Expression of nephrin and GM3 in early phase of anti-nephrin antibody-induced podocytopathy mice

A: Schedule for podocytopathy prevention test using valproic acid (VPA). **B**: Immunofluorescence staining images of glomeruli on day 1 after anti-nephrin antibody administration. Vehicle (Control), valproic acid (VPA) administered mice (VPA), 1.5 mg of anti-nephrin antibody (anti-Nphs Ab) induced podocytopathy (Ab-PDCP), and VPA+1.5 mg of anti-Nphs Ab induced podocytopathy (VPA+Ab-PDCP). Scale bars: 20 µm. Nephrin (green) and GM3 (red) merged areas (yellow) highlighted in enlarged images.

Supplementary Figure S1

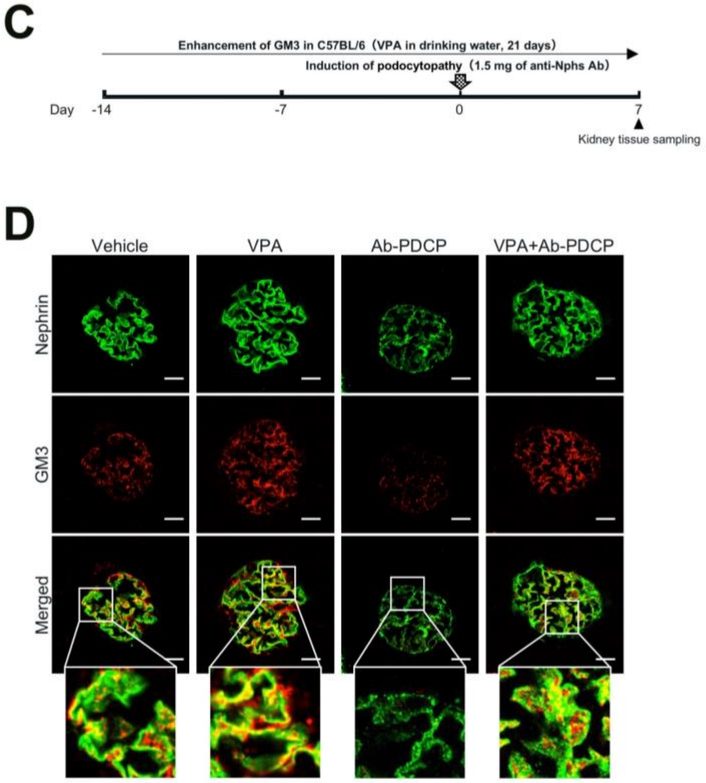

(continued) Supplementary Fig. S1. Expression of nephrin and GM3 in early phase of anti-nephrin antibody-induced podocytopathy mice

C: Schedule for podocytopathy prevention test using valproic acid (VPA). **D**: Immunofluorescence staining images of glomeruli on day 7 after anti-nephrin antibody administration. Vehicle (Control), valproic acid (VPA) administered mice (VPA), 1.5 mg of anti-nephrin antibody (anti-Nphs Ab) induced podocytopathy (Ab-PDCP), and VPA+1.5 mg of anti-Nphs Ab induced podocytopathy (VPA+Ab-PDCP). Scale bars: 20 µm. Nephrin (green) and GM3 (red) merged areas (yellow) highlighted in enlarged images.

Supplementary Figure S1

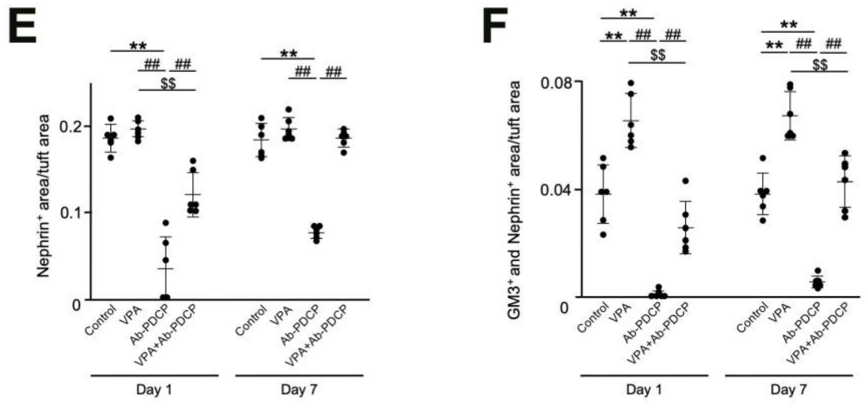

(continued) Supplementary Fig. S1. Expression of nephrin and GM3 in early phase of anti-nephrin antibody-induced podocytopathy mice

E: Scatter diagram showing nephrin fluorescence areas/tuft areas and **F**: GM3 and nephrin fluorescence areas/tuft areas of **B** and **D**. Twenty glomeruli per mouse were analyzed in each dot. $^{**}P$<0.01 vs. control, $^{\#\#}P$<0.01 vs. Ab-PDCP, $^{\$\$}P$<0.01 vs. VPA, $^{\dagger\dagger}P$<0.01 vs. day 1 in Ab-PDCP. Vehicle (Control), valproic acid (VPA) administered mice (VPA), 1.5 mg of anti-nephrin antibody (anti-Nphs Ab) induced podocytopathy (Ab-PDCP), and VPA+1.5 mg of anti-Nphs Ab induced podocytopathy (VPA+Ab-PDCP). Statistical analyses were performed from mice (n=6) in each group.

Supplementary Figure S2

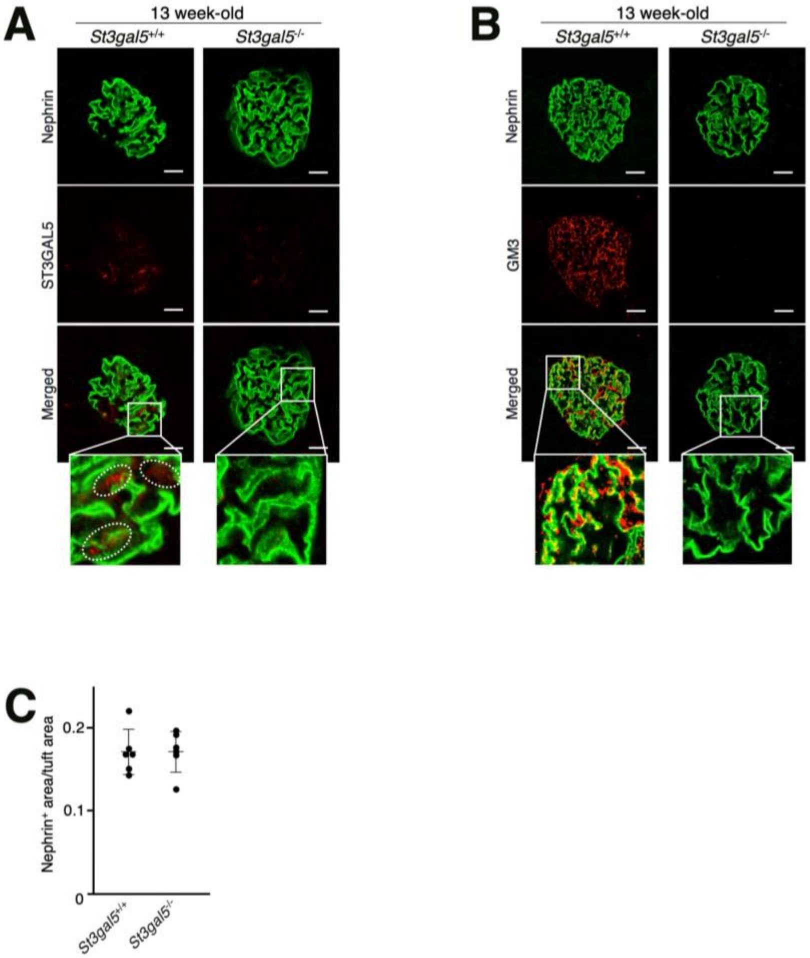

Supplementary Fig. S2. Expression of nephrin and ST3GAL5, or GM3 in wild-type (*St3gal5*$^{+/+}$) mice and GM3 synthase gene knockout (*St3gal5*$^{-/-}$) mice glomeruli

Immunofluorescence staining of glomeruli in *St3gal5*$^{+/+}$ *(*wild-type) and *St3gal5*$^{-/-}$ mice (both 13 weeks-old). **A**: Nephrin (green), ST3GAL5 (red) and merged florescence images of the glomeruli in both mice. **B**: Nephrin (green), GM3 (red) and merged florescence images of the glomerular of both mice. Scale bars: 20 µm. Nephrin and ST3GAL5, or GM3 merged areas (yellow) highlighted in enlarged images. **C**: Scatter diagram showing nephrin+ area/tuft area. Twenty glomeruli per mouse were analyzed in each dot, and each group were from n=6. **P*<0.05 vs. *St3gal5*$^{+/+}$.

Supplementary Figure S3

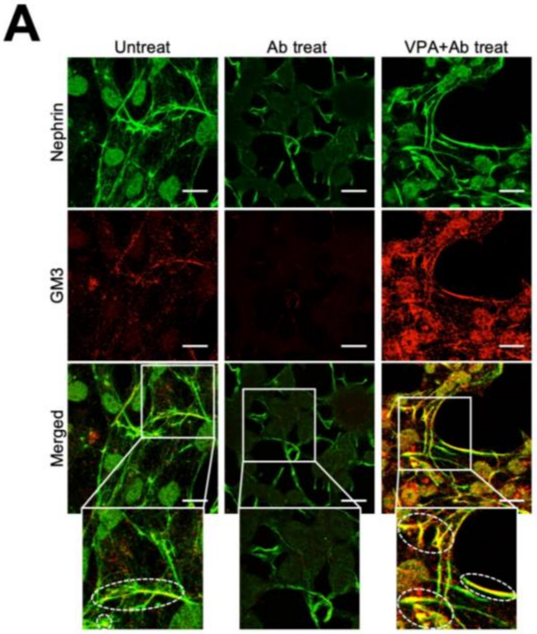

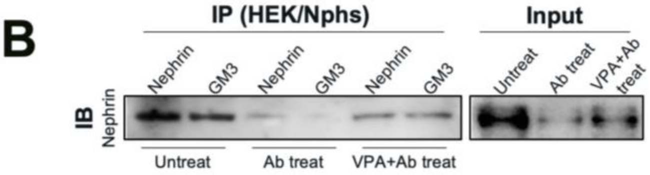

Supplementary Fig. S3. Influence of anti-nephrin antibody and valproic acid on HEK/Nphs cells and interaction of GM3 and nephrin

A: Immunofluorescence staining images of nephrin (green) and GM3 (red) in various treated HEK/Nphs cells. Areas highlighted by a white dashed line show merged areas (yellow) of nephrin and GM3 in enlarged images. Scale bars: 10 μm. **B**: Immunoprecipitation (IP) analysis using cell lysate of various treated HEK/Nphs. Untreated (Untreat), anti-Nphs Ab treated (Ab treat), preVPA+anti-Nphs Ab treated (VPA+Ab treat).

Supplementary Figure S4

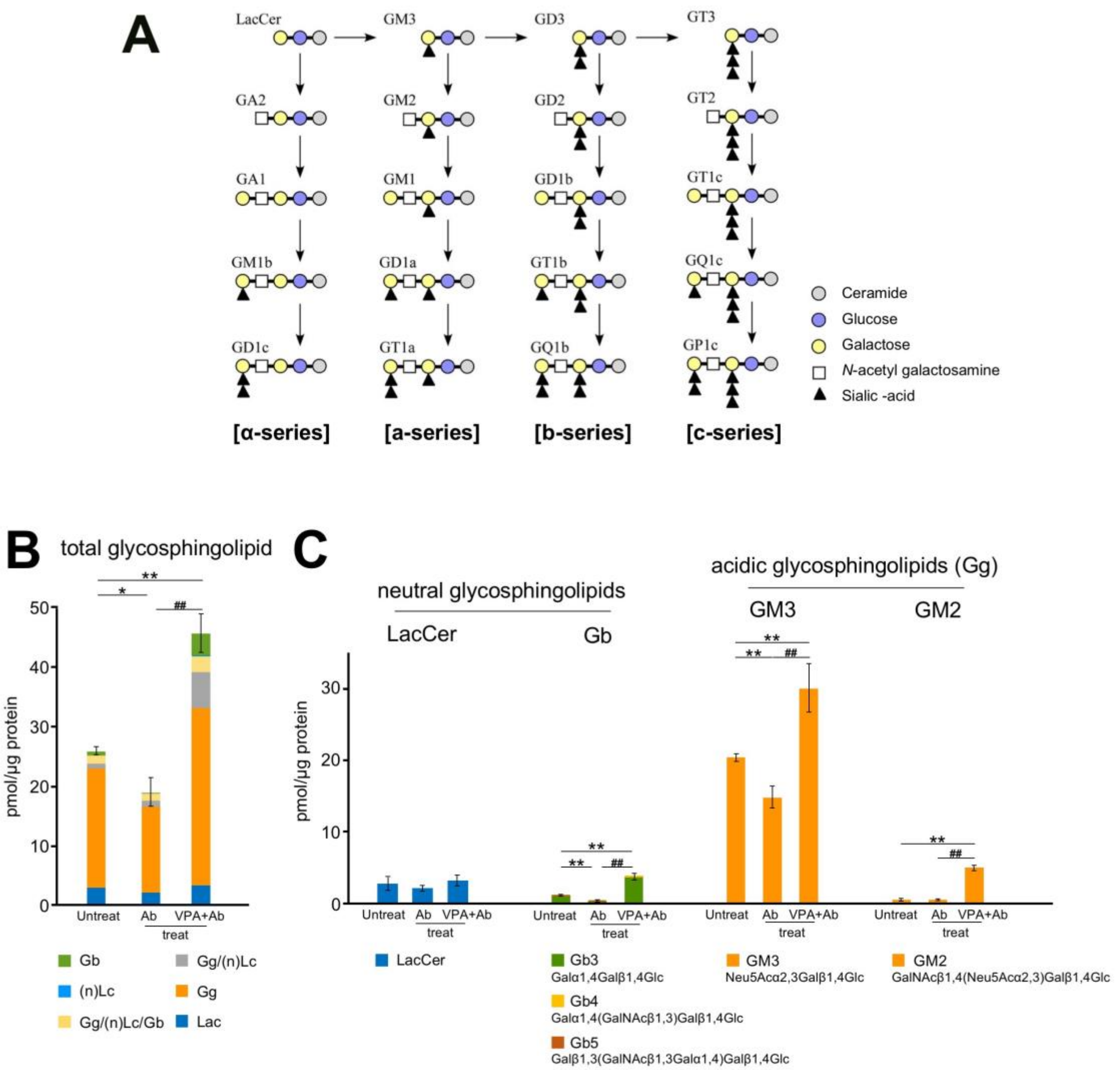

Supplementary Fig. S4. MALDI-TOF MS analysis of nephrin and GM3 in the cell membrane of HEK/Nphs

A: Biosynthetic pathways of ganglio-series of gangliosides. **B:** Amount of total glycosphingolipid in various treated HEK/Nphs cells. Each bar comprises the following; Gb: globo-series of gangliosides, (n)Lc: (neo)lacto-series of gangliosides, Gg/(n)Lc/Gb: ganglio-series of gangliosides/(neo)lacto-series of_gangliosides/globo-series of gangliosides, Gg/(n)Lc: ganglio-series of gangliosides/(neo)lacto-series of gangliosides, Gg: ganglio-series of gangliosides, Lac: Lac-series of gangliosides. **C:** Amount of neutral glycosphingolipids (LacCer, Gb) and acidic glycosphingolipids (GM3 and GM2) in various treated HEK/Nphs cells. Gb bar comprises the following; Gb3, Gb4, Gb5. $^{**}P<0.01$, $^{*}P<0.05$ vs. Untreat, $^{\#\#}P<0.01$ vs. Ab treat.

Supplementary Figure S4

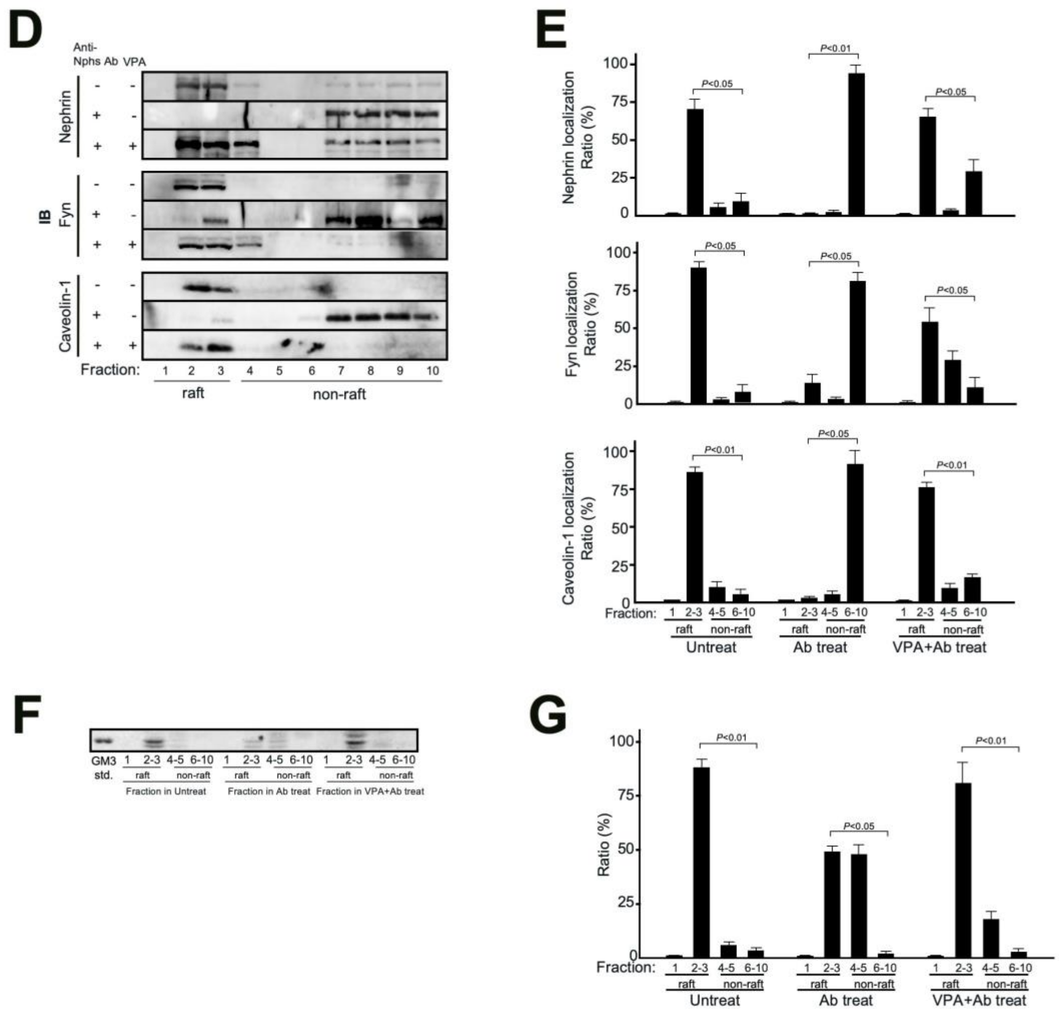

(continued) Supplementary Fig. S4. MALDI-TOF MS analysis of nephrin and GM3 in the cell membrane of HEK/Nphs

D: The distribution of each protein in the cell membrane fraction was determined by Western blot analysis. Fraction 1-3 and Fraction 4-10: raft fractions and non-raft fractions of various treated HEK/Nphs cells, respectively. Caveolin-1: raft marker protein. **E**: Bar graph representation of the results from **D**. **F**: Distribution of GM3 in the same cell membrane fraction as **D** exhibited by HPTLC with orcinol staining. Fraction 1-3 and Fraction 4-10: raft fractions and non-raft fractions of various treated HEK/Nphs cells, respectively. **G**: Bar graph representation of the results from **F**.

Supplementary Figure S5

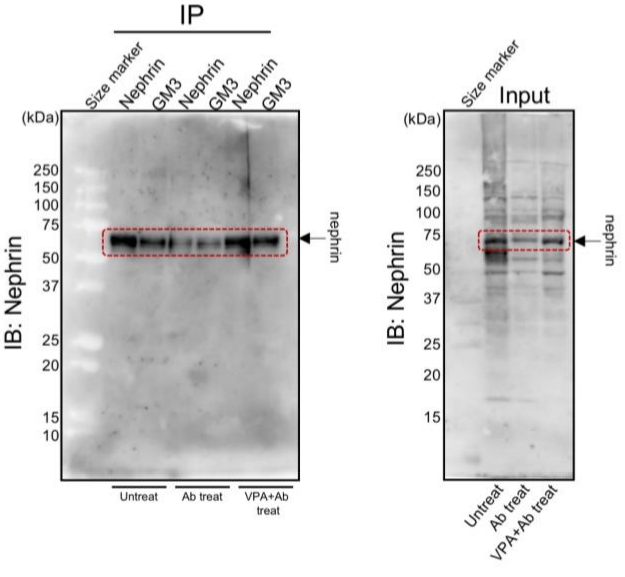

Supplementary Fig. S5.
Full length gel images of IP and Input of Figure 5.

Supplementary Figure S6

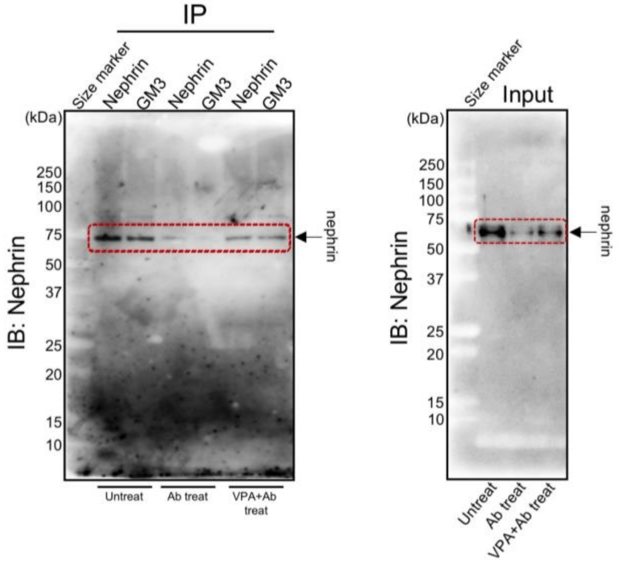

Supplemental Fig. S6.
Full length gel images of IP and Input of Supplementary Fig. S3.

Supplementary Figure S7

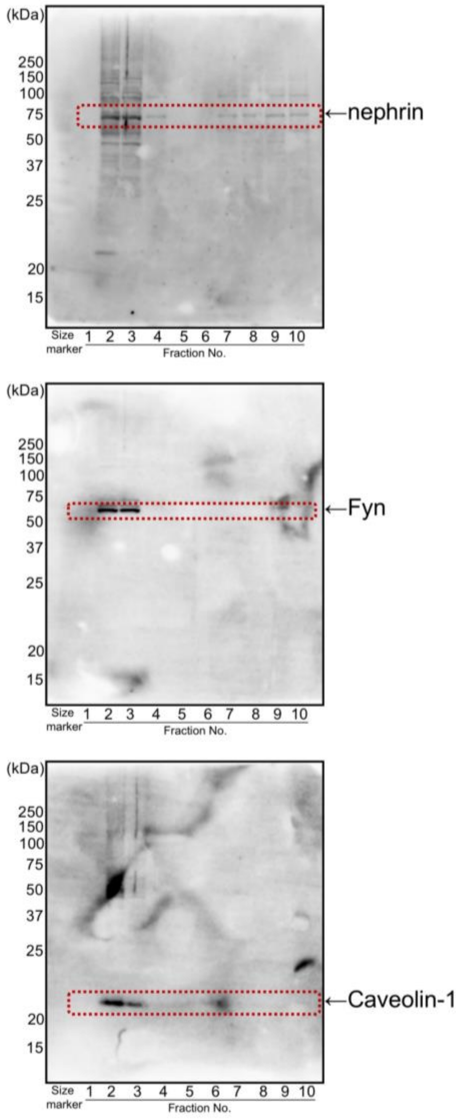

Supplemental Fig. S7 (Anti-Nphs Ab(-), VPA(-)).
Full length gel images of Supplementary Fig. S4 (HEK/Nphs_untreated).

Supplementary Figure S8

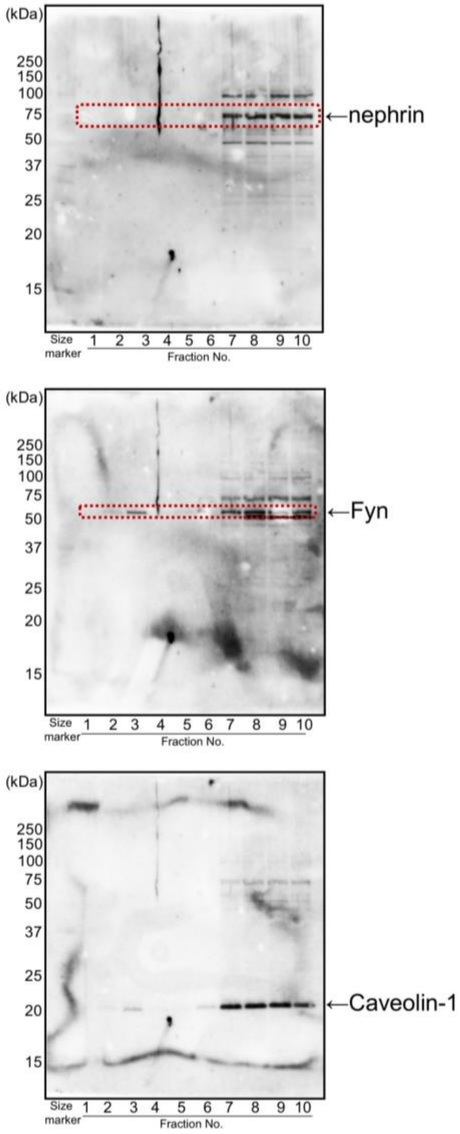

Supplemental Fig. S8 (Anti-Nphs Ab (+), VPA(-)).
Full length gel images of Supplementary Fig. S4 (HEK/Nphs_anti-Nphs Ab treated).

Supplementary Figure S9

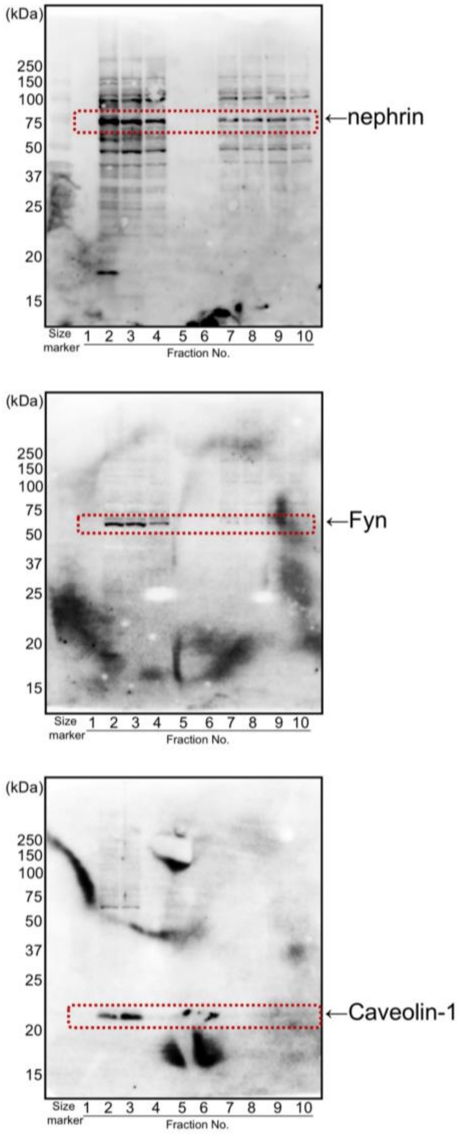

Supplemental Fig. S9 (Anti-Nphs Ab(+), VPA(+))**.**
Full length gel images of Supplementary Fig. S4 (HEK/Nphs_anti-Nphs Ab plus VPA treated).

Supplementary Figure S10

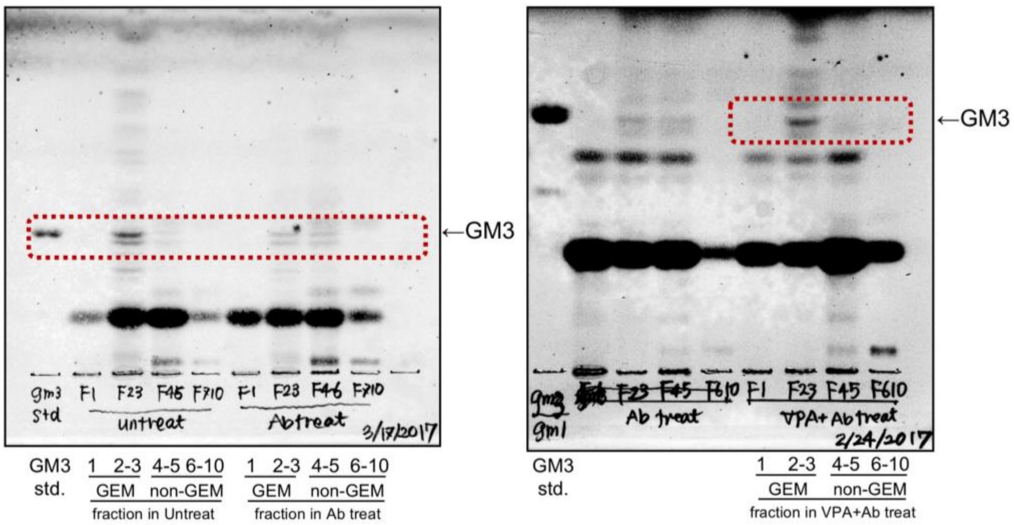

Supplemental Fig. S10.

Full length HPTLC plate images of Supplementary Fig. S4.

We could not do the development of long width HPTLC plate, because we do not have enough width development glass chamber. Therefore, we performed the development twice.

Supplementary Results

Supplementary Result S1 Expression of nephrin and GM3 in the early phase of anti-nephrin antibody-induced podocytopathy mice

To examine the expression level of nephrin and GM3 in glomeruli on day 1 and day 7 after 1.5 mg of anti-nephrin antibody induced podocytopathy and VPA pre-administration podocytopathy mice, immunofluorescence staining was performed. On day 1 after antibody administration in podocytopathy mice the expression levels of both nephrin and GM3 were clearly decreased, but on day 7 expression GM3 showed a slight recovery (Supplementary Fig. S1). In addition, there were no significant differences in the number of podocytes at both day 1 and day 7 of podocytopathy and VPA pre-administration podocytopathy mice compared with vehicle (Supplementary Fig. S1). These results suggested only molecules expressed on the slit diagram were affected in podocytopathy mice.

Supplementary Result S2 Influence of anti-nephrin antibody and VPA on HEK/Nphs cells

Expression of GM3, nephrin and F-actin bundle assembly in HEK/Nphs cells were compared under various treatment conditions (Supplementary Fig. S2A). As a result, after anti-Nphs Ab treatment GM3 was barely detectable and the levels of nephrin and F-actin assembly decreased. However, VPA treatment elicited an increase in the level of nephrin, GM3 and F-actin assembly despite anti-Nphs Ab treatment akin to that seen in VPA only treated cells. The results by immunocytochemistry also indicated a correlation with the results of anti-Nphs Ab-induced podocytopathy mice.

Next, we performed exhaustive analysis of the expression of all glycosphingolipid-glycans in HEK/Nphs cells were performed using Glycoblots and Tandem-MALDI-TOF/MS [6-9] (Supplementary Fig. S3B-C). The molecular species were analyzed and quantified using various treated HEK/Nphs cells. The amount of total Gg, especially GM3 ($(Hex)_2(Neu5Ac)_1$), in anti-Nphs Ab treated cells decreased compared with untreated cells. By contrast, VPA+anti-Nphs Ab treated cells displayed an increase in the amount of total Gg expression compared to untreated cells. Thus, VPA treatment elicited an increase in the level of total Gg, especially GM3 ($(Hex)_2(Neu5Ac)_1$), even though the cells were treated with anti-Nphs Ab at the same time. There was, however, no significant change in the levels of Lc and total Gb under the various treatment conditions.

Furthermore, we examined whether the effect of VPA in preventing podocytopathy was solely due to the elevated levels of GM3.

Supplementary References

1 Takeuchi, K. *et al.* New Anti-Nephrin Antibody Mediated Podocyte Injury Model Using a C57BL/6 Mouse Strain. *Nephron* **138**, 71-87, doi:10.1159/000479935 (2018).

2 Shankland, S. J., Pippin, J. W., Reiser, J. & Mundel, P. Podocytes in culture: past, present, and future. *Kidney Int* **72**, 26-36, doi:10.1038/sj.ki.5002291 (2007).

3 Uemura, S., Yoshida, S., Shishido, F. & Inokuchi, J. The cytoplasmic tail of GM3 synthase defines its subcellular localization, stability, and in vivo activity. *Mol Biol Cell* **20**, 3088-3100, doi:10.1091/mbc.E08-12-1219 (2009).

4 Kawashima, N., Yoon, S. J., Itoh, K. & Nakayama, K. Tyrosine kinase activity of epidermal growth factor receptor is regulated by GM3 binding through carbohydrate to carbohydrate interactions. *J Biol Chem* **284**, 6147-6155, doi:10.1074/jbc.M808171200 (2009).

5 Liang, Y. J. *et al.* Differential expression profiles of glycosphingolipids in human breast cancer stem cells vs. cancer non-stem cells. *Proc Natl Acad Sci U S A* **110**, 4968-4973, doi:10.1073/pnas.1302825110 (2013).

6 Ishibashi, Y. *et al.* A novel endoglycoceramidase hydrolyzes oligogalactosylceramides to produce galactooligosaccharides and ceramides. *J Biol Chem* **282**, 11386-11396, doi:10.1074/jbc.M608445200 (2007).

7 Fujitani, N. *et al.* Qualitative and quantitative cellular glycomics of glycosphingolipids based on rhodococcal endoglycosylceramidase-assisted glycan cleavage, glycoblotting-assisted sample preparation, and matrix-assisted laser desorption ionization tandem time-of-flight mass spectrometry analysis. *J Biol Chem* **286**, 41669-41679, doi:10.1074/jbc.M111.301796 (2011).

8 Furukawa, J. *et al.* Comprehensive approach to structural and functional glycomics based on chemoselective glycoblotting and sequential tag conversion. *Anal Chem* **80**, 1094-1101, doi:10.1021/ac702124d (2008).

9 Furukawa, J. *et al.* Quantitative GSL-glycome analysis of human whole serum based on an EGCase digestion and glycoblotting method. *J Lipid Res* **56**, 2399-2407, doi:10.1194/jlr.D062083 (2015).
